# Portable hand‐held bioprinters promote in situ tissue regeneration

**DOI:** 10.1002/btm2.10307

**Published:** 2022-03-10

**Authors:** Zahra Pazhouhnia, Nima Beheshtizadeh, Mojdeh Salehi Namini, Nasrin Lotfibakhshaiesh

**Affiliations:** ^1^ Department of Tissue Engineering School of Advanced Technologies in Medicine, Tehran University of Medical Sciences Tehran Iran; ^2^ Regenerative Medicine group (REMED) Universal Scientific Education and Research Network (USERN) Tehran Iran

**Keywords:** in situ regeneration, portable bioprinter, regenerative medicine, tissue engineering

## Abstract

Three‐dimensional bioprinting, as a novel technique of fabricating engineered tissues, is positively correlated with the ultimate goal of regenerative medicine, which is the restoration, reconstruction, and repair of lost and/or damaged tissue function. The progressive trend of this technology resulted in developing the portable hand‐held bioprinters, which could be used quite easily by surgeons and physicians. With the advent of portable hand‐held bioprinters, the obstacles and challenges of utilizing statistical bioprinters could be resolved. This review attempts to discuss the advantages and challenges of portable hand‐held bioprinters via in situ tissue regeneration. All the tissues that have been investigated by this approach were reviewed, including skin, cartilage, bone, dental, and skeletal muscle regeneration, while the tissues that could be regenerated via this approach are targeted in the authors' perspective. The design and applications of hand‐held bioprinters were discussed widely, and the marketed printers were introduced. It has been prospected that these facilities could ameliorate translating the regenerative medicine science from the bench to the bedside actively.

**Abbreviations**
AM: additive manufacturing
ADSCs: adipose‐derived stromal/stem cells
AG: agarose
BMP‐2: bone morphogenetic protein 2
COL1: collagen type I
CT: computed tomography
ECM: extracellular matrix
FDM: fused deposition modeling
GelMA: gelatin methacryloyl
HGM: host–guest macromer
hBMSCs: human bone marrow‐derived mesenchymal stem cells
HUVEC: human umbilical vein endothelial cells
hADSCs: human‐derived mesenchymal stem cells
HA: hyaluronic acid
HAMa: hyaluronic acid–methacrylate
HAp: hydroxyapatite
IPFP: infrapatellar fat pad
ICRS: international cartilage repair society
MRI: magnetic resonance imaging
MSCs: mesenchymal stem cells
OA: osteoarthritis
POC: point‐of‐carry
PCL: polycaprolactone
PEGDA: polyethylene (glycol) diacrylate
PEO: polyethylene oxide
RP: rapid prototyping
SLS: selective laser sintering
3D: three‐dimensional
TE: tissue engineering
VEGF: vascular endothelial growth factor
VML: volumetric muscle loss
WHO: World health organization

## 1 INTRODUCTION

### 1.1 Bioprinting

The exceptional capabilities of additive manufacturing (AM) technology, which was emerged in the late 90s, such as removing structural limitations and the breadth of printable materials, shortly established itself as a significant component of the fourth industrial revolution.^1^ In the meantime, relying on the printing layer‐by‐layer, AM technology, went beyond the industry in the late 90s. It introduced itself as a revolutionary technology in the world of medicine and medical engineering.^2^ Since human organs' have quite a complex geometric structure and are unique to each person, bioprinting technology relies on layer‐by‐layer printing, would be beneficial in fabricating tissues and in near future organs. It has possibility of producing each person's organ perfectly fit the individual's physiological condition.^2^

People have always exposed to a variety of diseases and accidents.^3^ They have sometimes plagued by disability obstacles and a variety of diseases, such as cancer, osteoporosis, bone fracture, and skin burns. Physicians typically need testing and experience to diagnose and treat diseases, which usually gained through testing on volunteer patients or laboratory animals. In the event of a limb defect and the need to replace it, there are always long queues of patients waiting for a transplanted organ to arrive from a brain‐dead patient.^4^ With the advancements in genetics and the ability of stem cells to multiply and differentiate, in vitro, medicinal science has entered a new phase, and it has become possible for humans to get rid of suffering from some diseases and disabilities.^5^

Bioprinting technology has become with the help of medical science to promote human health in some related areas, such as drug development, disease modeling, and production of various tissues for transplantation.^6^ Already, the cost of medical research is pretty high, and the number of available samples is generally less than required, which has slowed down the research process, which imposes a high cost on research centers economically.^7^ This technology has made it possible to print part of an organ needed for research and perform a variety of experiments on it. Also, for patients whose part of the organ is diseased or defective, a suitable replacement for that organ can be made by bioprinting method and implanted into the body of the patient.^8^

Ultimately, the main goal of this technology in the near future is to build a living organ and transplant it into patients' body. Also, regarding using bioprinting technology, the costs of drug production and research on diseases would be significantly reduced.^9^ Consequently, the development in this area would take a primary tool for research and treatment, from bench to the bedside.^10^ The design and manufacture of bioprinters with various applications have considered by research and industrial centers.

Today, more than 30 active companies in the field of bioprinting are operating in developed countries, some of which have proprietary technology and have taken significant steps to print live organ.^11^ Massive investment in this field shows the great importance of the issue that three‐dimensional (3D) bioprinting could empower the economics of regenerative medicine.^12^

Bioprinting, applying the principles of rapid modeling technology, is a more promising solution for tissue engineering (TE). Utilizing this technology, researchers can create a variety of biological materials, including cells and multiple tissue scaffolds, using 3D printing.^13^ A new method for body tissue reconstruction emerges from progress in three areas, including rapid prototyping (RP) techniques, smart polymers, and cellular adhesion.^14^ Eventually, what results from the ideal combination of these three components is tissue 3D printing.^15^

Using the concepts of evolutionary biology, the principles of bioengineering, biomaterial engineering, and computing methods, bioprinting technology seek to create alternative tissues and organs to solve human medical dilemmas.^16^ Perhaps it can be said that what is more considered in this TE strategy than other traditional and even modern methods, such as computer‐based TE, is attention to the cellular principles. This method tries to rely on what is in the model. The capture of a natural tissue occurs within the body and, with the utmost effort to comprehensively biologically stimulate the tissues. Organ printing technology has multiple roots and based on the efforts of several interdisciplinary teams of various disciplines and scientists. Bioprinting utilizes inkjet cartridges loaded with cells and the proper ink, which are dispensed onto a substrate along with biomaterial.^17^

In general, the printing procedure consists of three consecutive stages, including preprinting, printing, and postprinting. In the preprinting stage, the initial large‐scale design of the target organ designed by computer simulation based on images obtained from the patient's damaged tissues.^18^ Digitally reconstructed pictures of that organ from magnetic resonance imaging (MRI) or computed tomography (CT), or mathematical models, can be employed in this way. The printer builds up a 3D structure layer‐by‐layer until the desired result is achieved. The tissues produced at this stage are not yet functional. In the postprinting phase, the structures created by the appropriate bioreactors are given nonstop with biomechanical conditions.^19^

Although bioprinters have the potential to save several lives in the future, numerous difficulties remain. For example, printed structures may be fragile and unable to maintain their shape when transferred to an appropriate location on the body. Additionally, tissues and organs are complicated and contain a variety of distinct cell types that are tightly controlled. Finally, present approaches are constrained by their reliance on particular materials, a narrow viscosity range, and a lack of precision. Any approach has the risk of causing harm to cells and other printing materials. That is why researchers and engineers are trying to solve issues and design new bioprinters with unique capabilities.^20^

### 1.2 Tissue regeneration

Recent advances in stem cell‐based technology have distinguished TE among regenerative medicine sciences.^21^ Regenerative medicine has guided the treatment of diseases through gene therapy, cell transfusions, or other minimally invasive procedures that avoid replacing the entire organ.^22^ TE is a new field of regenerative medicine that helps to repair or restore tissue defects. Also, it could be creating an artificial environment for cell growth and stimulate tissue regeneration.^23^ The ultimate objective of TE is to generate functioning cells, tissues, and organs in order to repair, replace, or augment biological function lost to illness and damage.^24^ Additionally, it is one of the most promising strategies for overcoming the lack of appropriate organs for transplantation.^21^, ^25^ Lack of organ transplants can be the most valuable practical reason for the development of TE.

In acute wounds, the wound healing process naturally heals, while in chronic or incurable wounds, due to an internal or external wound agent's intervention, it stops at one of the healing stages, and the wound healing process slows down or disrupted.^26^ As soon as the wound created, the body has the task of starting and continuing several reactions simultaneously, including preventing bleeding, preventing the invasion of bacteria and microorganisms, removing dead tissue and foreign bodies from the wound site, and producing new tissue in the wound area.^27^ TE could create an artificial environment for cell growth and stimulate tissue regeneration.^28^

A tissue is made up of 3D units that are repeated at a size of 100–1000 nm in vivo environment.^29^ In order to coordinate multicellular activities, generate mechanical qualities, and integrate with other organ systems via microcirculation, this repeating tissue's 3D structure is essential. Structural support is provided by the scaffolds during tissue restoration in order to promote 3D cell evolution.^29^ Tissues are also influenced by the local cellular environment. Biochemical, cellular, and physical catalytic signaling pathways involved in cellular destiny events such as differentiation, proliferation, migration, and death are all dependent on the microbiological environment (10 m). Micro‐ and macro‐scale levels should be addressed in order to fabricate functional scaffolds successfully.

Today, researchers are trying to design scaffolds with multiple capabilities, since scaffolding, must be able to guide tissue formation and even prevent possible damage, in addition to providing temporary structural support to the tissue being formed.^30^ Optimal scaffolding design and fabrication methods should create porous structures that allow cell activity, material translocation and angiogenesis. These porous structures must have pores that are first sized and second interconnected to each other.

In conventional scaffolding methods such as solvent casting and salt washing, gas foaming, freeze‐drying, and fuzzy separation, it is impossible to precisely control the shape, size, and relationship of pores to each other. Also, the widespread use of organic solvents in many of these methods can lead to adverse effects on biological tests' results in vitro and inflammatory responses in the body. On the other hand, it is impossible to reproduce the scaffolds in the mentioned methods so that all samples' structures and properties are the same.

The conventional and RP methods of 3D scaffold production are classified into two categories, each creating a different type of scaffold. Standard scaffolding fabrication techniques include the production of porous polymer structures such as cell adhesion substrates; however, complex structures with tunable microscale and macroscale are challenging to create using conventional procedures.

Traditional scaffold fabrication methods such as solvent casting/particulate leaching are intended to define the shape and pore size of the scaffold. However, they are mostly limited to the prior scaffold internal design or connectivity of the void space in 3D cell cultures. Using a solvent and salt particles of a certain size, the polymer solution is dissolved in the salt matrix, which is then submerged in water, and the salt leaches away to form a structure with high porosity. Solvent casting with particle leaching is only suitable for thin membranes of thin‐walled 3D specimens; otherwise, the soluble particles cannot be separated from the salt matrix.^31^ This approach produces scaffolds with a porosity of between 50% and 90%. The high porosity and capacity to fine‐tune the pore size of the scaffold created by this method make it ideal for the formation and proliferation of 3D cells. The number of pore networks between layers of porous sheets is limited, limiting its suitability for an application. Hazardous solvents that take a long time (days or weeks) to evaporate are used in this method.^31^

Moreover, freeze‐drying or lyophilization is a method in which a synthetic polymer that has been dissolved in a suitable solvent is used to dry a sample.^32^ A solid solvent is evaporated by sublimation after dissolving a polymer solution. This leaves a solid scaffold with multiple linked pores. A fence of matter surrounds the ice in the ice phase, allowing the solutes to be isolated from one another. The scaffolds are created after subsequent drying, and the macroporosity is accomplished by simply dissolving and freeze‐drying the initial empty area occupied by ice crystals. The advantage of this technology is that it does not use high temperatures, which could reduce the activity of biological factors that are incorporated. In addition, the freezing process can be modified to alter the pore size. Experts claim that this technology is flourishing in manufacturing scaffolds for a wide range of applications.^33^ However, despite the fact that this method is frequently used in producing the scaffolds, it still has several drawbacks, including high‐energy consumption, a protracted timescale, and the use of cytotoxic solvents. As a solution to these issues, the researchers experimented with different freezing temperatures (ranging from 10° to 70° Fahrenheit) and the addition of an additional annealing stage.^33^

The use of water and ice crystals rather than an organic solvent during scaffold creation makes freeze‐drying a more appropriate method for biomedical applications; however, this method is challenged in the fabrication of hierarchically organized scaffolds in biomedicine, such as vascular systems. The constructed scaffold must also be rinsed numerous times to eliminate the cytotoxic solvents used to mix the polymer and reduce cell death.

In order to deal with the use of high temperatures and organic cytotoxic solvents, the gas foaming technique has been developed. This technique uses comparatively inert gas foaming agents such as carbon dioxide and nitrogen to press a biologically degradable polymer model with water until it is saturated or full of gas bubbles.^34^ A sponge‐like structure with pore sizes ranging from 30 to 700 μm and porosity of up to 85% can be created using this method.^34^ In some cases, the product produced may have a solid polymeric skin or a closed pore structure.

In nanofibrous scaffold development, electrospinning is crucial for producing fibers from a solution using electricity.^35^ Utilizing electrospinning, a high‐voltage charge causes droplets on the spinneret to erupt, causing them to stretch and elongate. The homogenous distribution of pores is challenging to achieve using electrospinning, despite the fact that it is an easy and quick approach for making nanofibrous scaffolds. As a result, only limited uses in biomedicine have been found for this technique.^35^

In contrast to traditional scaffolding methods, RP systems use powder, liquid, and sheet materials to create items. Layer‐by‐layer, an RP machine can create objects made of wood, ceramic, plastic, or metal from a computer‐generated model with tiny horizontal cross sections.^36^ The RP scaffold construction methodology allows for fine spatial control over the polymer structure to overcome some of the difficulties inherent in standard production methods. These approaches allow for the creation of personalized and patient‐specific scaffolds that are suited for the tissues and organs in question.

TE has a wide range of possible applications for RP scaffold creation. Micro‐ and macro‐scale features can be controlled independently to create multicellular structures that are necessary for complicated tissue functions. To begin with, the construction of 3D circulatory beds will provide support for much larger tissue formations than would otherwise be conceivable. Combining clinical imaging data with 3D fabrication processes allows for the creation of personalized scaffolds and mass production of the scaffold designs.

Some of the most common RP processes include 3D printing, fused deposition modeling (FDM), selective laser sintering (SLS), and stereolithography. Stereolithography is a 3D printing technique that uses a thin layer of UV‐curable material to build up a solid object layer‐by‐layer.^37^ A stereolithography system consists of four essential parts, including a tank with a photosensitive liquid resin, a transportable constructed platform, a UV laser for radiating resin, and a dynamic mirror system.^37^ The process begins with a UV laser by depositing a layer of photosensitive liquid resin on the platform. The platform is then lowered vertically once the first layer has been set. Afterward, the process is repeated until a 3D scaffold is formed. It is then cleaned and postcured under UV light to remove any remaining resin. As a result, the waste associated with subtractive production methods is eliminated by this technology. Stereolithography is a robust biofabrication method that can be used in a wide range of fields, including biosensing, environmental remediation, drug discovery, and energy harvesting.^37^

Using computer‐controlled extrusion and deposition procedures, a solid polymer is melted and extruded onto the surface of a 3D object using the FDM technology.^38^ The scaffold is constructed from numerous layers of neighboring microfilaments. Thermoplastic biopolymers have been processed using FDM. Also, SLS uses a laser as a power source to sinter powdered materials defined by a 3D model. This method has been used to create a wide range of materials, including polymers, metals, and ceramics. Tools and practical prototype features can be built directly from computer models using 3D Printing. The powdered substance is applied in layers and selectively fused via inkjet printing, where the adhesive is produced. The unbound powder is removed from the layers, resulting in a complex 3D shape.^39^ This procedure can be used to manufacture ceramic, metal, and metal/ceramic composite parts.

3D polymer deposition is a novel TE fabrication process that enables scaffold structure microlevel precision control.^40^ Even though the scaffolds created by the 3D Printing technology are able to replicate the shape of the natural tissue and the mechanical features of the scaffold, they lack the ability to mimic the nanoscale ECM properties that are essential to the success of the procedure. Using material transfer techniques for producing a biological pattern and assembly of relevant materials, cells, molecules, tissues, or biodegradable biomaterials with a predetermined structure to perform some biological activities, bioprinting is a 3D printing technology. Using solvent‐free, aqueous‐based printing methods, it is now possible to print 3D scaffolds for transplantation with or without seeded cells.

Furthermore, bioprinting is a technique that uses cell development to enable individualized medication. 3D bioprinting technologies can currently be divided into acellular and cellular. It is possible to print a scaffold and biomaterial without a cell using acellular bioprinting technology. Compared to cell‐based bioprinting, acellular bioprinting provides a higher level of accuracy and greater shape complexity because it does not require the maintenance of cell viability during construction. It is possible to create living tissue constructs by integrating cells and other bioagents into the material during manufacture. This means that in order to build these structures, circumstances, and parameters must be optimized based on the presence or absence of living cells and biological components.

Autonomous self‐assembly, biomimicry, and mini‐tissue building blocks are only a few examples of 3D bioprinting methods. It is currently the most commonly used approach to deposit and pattern biological materials using micro‐extrusion, laser‐assisted printing, or inkjet printing. For example, inkjet bioprinting uses picolitre droplets of bioink to build 2D or 3D objects on a substrate without the need for touch. These approaches, for example, are ideal for a wide range of applications, including biomedical research, due to their low cost, rapid speed, and high cell viability (80%–90%). In addition, the approach has narrow material selectivity, frequent print head clogging, and difficulty retaining biological material in liquid conditions for droplet production.^41^

The capacity to precisely insert cells, biomaterials, and chemicals for tissue regeneration has increased interest in bioprinting technology.^42^ Additionally, this technique has been utilized to perform trials for 3D printing customized skin transplants for patients with extensive wound regions, muscle, and even micro stereolithography 3D printing to repair damaged nerve connections.^43^ The delivery of nutrients and oxygen to cells is a critical aspect of cell viability.

Angiogenesis is often the most critical and challenging part of a TE process. So far, various methods have used to supply tissue oxygen to facilitate angiogenesis, which has not been as effective because the rate of vascular formation within tissues is naturally prolonged.^44^ Due to the need to create branched vascular structures in printed tissues, the first and most crucial step is to print tubular structures. In practice, it is possible to create vascular tubes by placing the masses of endothelial cells together in three dimensions and connecting them in two directions, plane and vertical.^45^ Reproducibility and scalability of 3D bioprinter technologies allow us to build structures. Recently, the use of bioprinters has provided new possibilities for the development and application of vascular systems in the rehabilitation of different tissues.^46^

### 1.3 In situ bioprinting

In situ bioprinting is a novel technology that merges with portable bioprinting, so it has some facilities to improve the disease and accelerate healing.^47^ When compared to standard in vitro bioprinting, in situ bioprinting may be favored since de novo tissues are produced immediately on the predicted anatomical site in the real organism.^48^

In situ bioprinting may be described as the direct printing of bioinks to build or repair living tissues or organs at a fault location in a clinical context.^49^ Alternatively, the anatomical area where regeneration is needed in the body might be used as a printing site. Conventional 3D printing methods are often limited to flat surfaces, but this methodology intends to repair and replace damaged tissues with curved surfaces or more complex geometries.^48^ However, the safety and sterility of this technology must be thoroughly validated before it can be implemented in clinical settings. Because of the lack of expertise necessary for in situ bioprinting in the clinical environment, clinicians prefer to use off‐the‐shelf alternatives, such as implanting a prefabricated construct at the defect location.^50^ However, recent research has proven the enormous promise of this method, notably in the areas of skin, bone, and cartilage regeneration.

In general, in situ bioprinters are divided into two categories, including the robotic arm approach and hand‐held approach. There is less human intervention in the former one, and the system includes a three‐axis moving bioprinter. This device may have one or more print heads and may utilize a variety of bio‐inks and cross‐linking agents. Hand‐held bioprinters are a portable device equipped with a printing mechanism that enables the direct printing of biological materials and cells at the site of damage.^43^ Among the advantages of this type of bioprinters, convenient transportation, easy access to the damaged area due to the small size of the device, and the low cost of device manufacturing are the most significant parameters.^51^ Various studies were performed on the use of hand‐held bioprinters.^52^, ^53^

Due to the lack of the establishment of an artificial microenvironment, the in situ TE technique has an advantage over the conventional TE approach. A functioning tissue or organ can only be created in the absence of these biochemical and biophysical factors, which is why ex vivo techniques rely primarily on mimicking the original tissue milieu (which includes the requisite biochemical and biophysical signals). As a result, surgeons are able to use in situ bioprinting techniques, which entail either robotic arms or a portable device with a separate bioprinting unit that may be inserted into a patient's body to repair injured tissue.^54^ An in vivo bioreactor governs the development and maturity of the printed construct under this method. However, it is essential to keep the recipient organ immobilized during the printing process (e.g., the musculoskeletal system and craniofacial skeletal abnormalities, etc.). While in situ bioprinting has yet to reach its full potential, it is understandable to develop the technique for superficial organs. It is possible to print not just on horizontal surfaces but also to cover irregularly shaped tissue defects with a contour deposition approach using special robotic hands.

Additionally, in situ bioprinters have a practical application in the treatment of chronic wounds such as diabetic wounds, pressure ulcers, and burn wounds. The skin bio‐printer employs a cartridge‐based delivery method coupled to a portable XYZ plotting device. Cartridges are identical to those used in conventional inkjet printing. Each cell type is housed within a separate cartridge, much as different color inks are held within separate cartridges. The cartridge‐based architecture of the skin bio‐printer enables the direct printing of almost any cell type, macromolecule, or biomaterial in a wound region.^55^

Aside from the additional stages and equipment required, the in vitro printing of scaffolds also increases the danger of infection. The incision must be more significant for surgical reasons than the proposed procedure. The implantation location may also require the use of another adhesive media. This is not a crucial issue with current technology because printing takes place on‐site, there is no need for transportation, and the printed scaffold can cure right where it is placed. On‐site printing becomes inherently stable because the printer can construct an inherently stable form configuration at implantation. As a result, patients can be released from the hospital sooner, allowing for faster recovery, and the hospital saves money since fewer beds are needed due to lower bed occupancy.

## 2 DESIGN AND APPLICATIONS OF PORTABLE BIOPRINTERS

The ultimate purpose of emerging and introducing portable bioprinters is the ease of use and fast transportation in clinical conditions without the need for complex operator training. This technology is evolving at an incredible rate, which various institutes and companies are developing this technology with some innovations, with the common goal of accelerating the device's repair process and portability.

**FIGURE 1:**
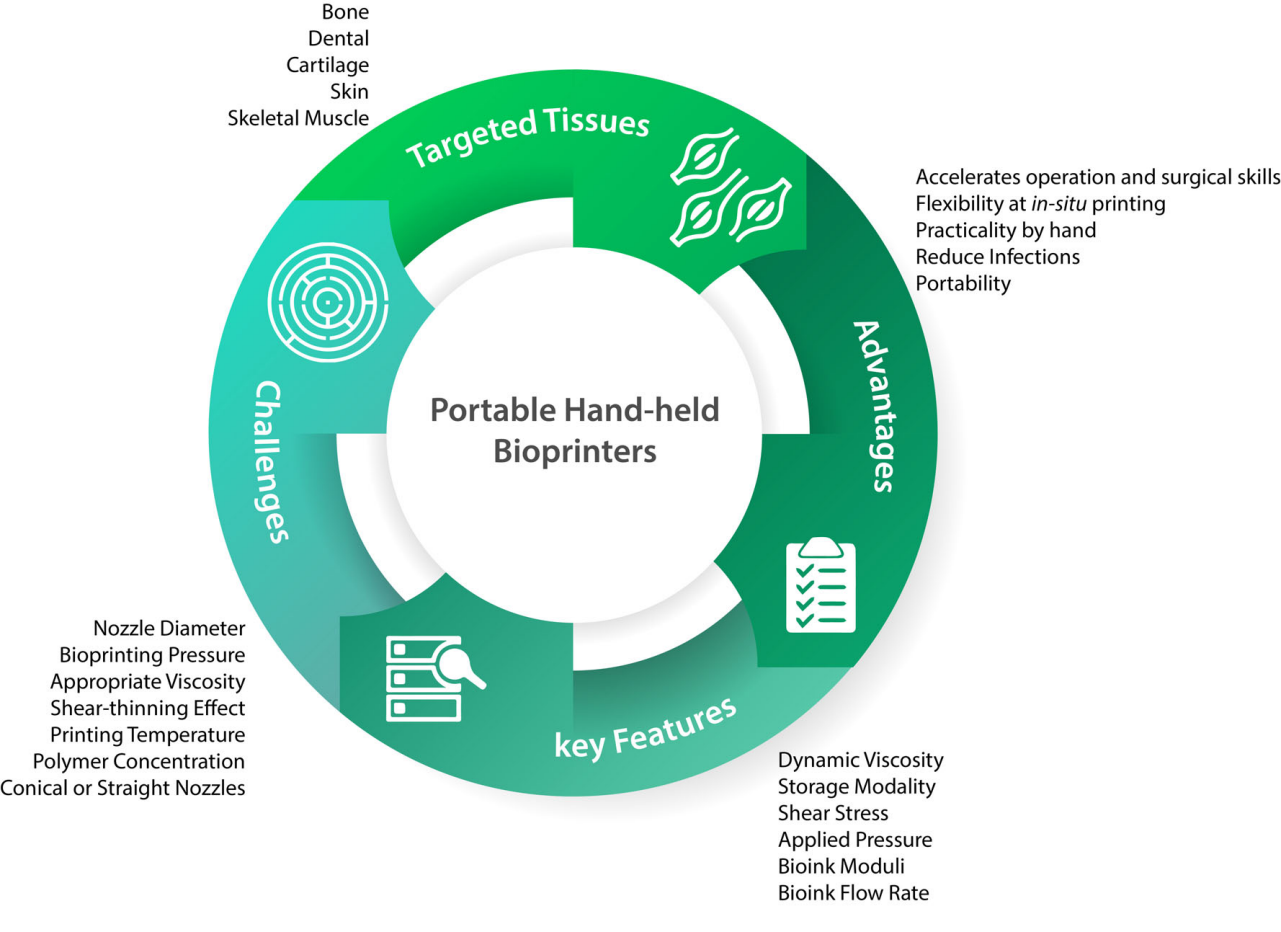
Advantages, challenges, key features, and targeted tissues of portable hand‐held bioprinters

Regarding the wound dressings in treating acute and chronic wounds, they suffer from tremendous challenges, including the need to be changed at multiple times, scar tissue, the possibility of infection at the wound site, and the reduction of angiogenesis for wound healing. To deal with these issues, new methods of treating via hand‐held bioprinters have entered the market, which has solved these issues considerably. Multiple features along with several challenges could be considered utilizing portable hand‐held bioprinters to regenerate multiple tissues (Figure 1).

### 2.1 The bioink flow‐rate in bioprinting

The physical properties of bioinks could be altered based on the concentrations of hydrogel and cellular components in the bioink. Extrusion printing aims to produce stable, stackable filaments in a repeatable and predictable process, as well as to understand the connections between bioink qualities and extrusion parameters; hence, the desired output may be achieved. Hydrogels made from alginate, alginate/gelatin, and other materials that give repeatable and dependable outcomes with excellent form fidelity are commonly used to represent extrusion dynamics. However, while these hydrogels have improved our knowledge of the elements that affect bioprinting, they have limitations that do not applied to other materials.

Also, several key features are needed for the extrusion bioprinting process, including dynamic viscosity, loss modulus, and storage modality.^56^ When shear stress and applied pressure are kept under control, the viability of cells is sustained. As a result, it is critical to know how flow rate, shear stress, applied pressure, moduli, and viscosity are all connected. Most bioinks are not modeled according to Newtonian fluids, so their properties cannot be compared to those of classical fluids.^57^ A direct and linear relationship between an applied force and the resultant flow results in a constant viscosity at a given temperature and air pressure for Newtonian fluids, which is beneficial for simplifying calculations and estimations or for materials having qualities similar to Newtonian.^58^ Since the viscosity of non‐Newtonian fluids varies according to the exerting force, the relationship between the exerting force and the fluid flow is more complicated. For a Newtonian fluid in laminar flow conditions, the Hagen–Poiseuille equation (Equation 1) that relates the volumetric flow rate (Q) to the pressure applied (P) to a nozzle length (L) and diameter (d), and viscosity (η), could be used to estimate the bioink viscosity.^59^

(1) $$Q=\frac{\pi ∆pd^{4}}{128\;\mathrm{L\eta}}$$

### 2.2 Portability and flexibility

**FIGURE 2:**
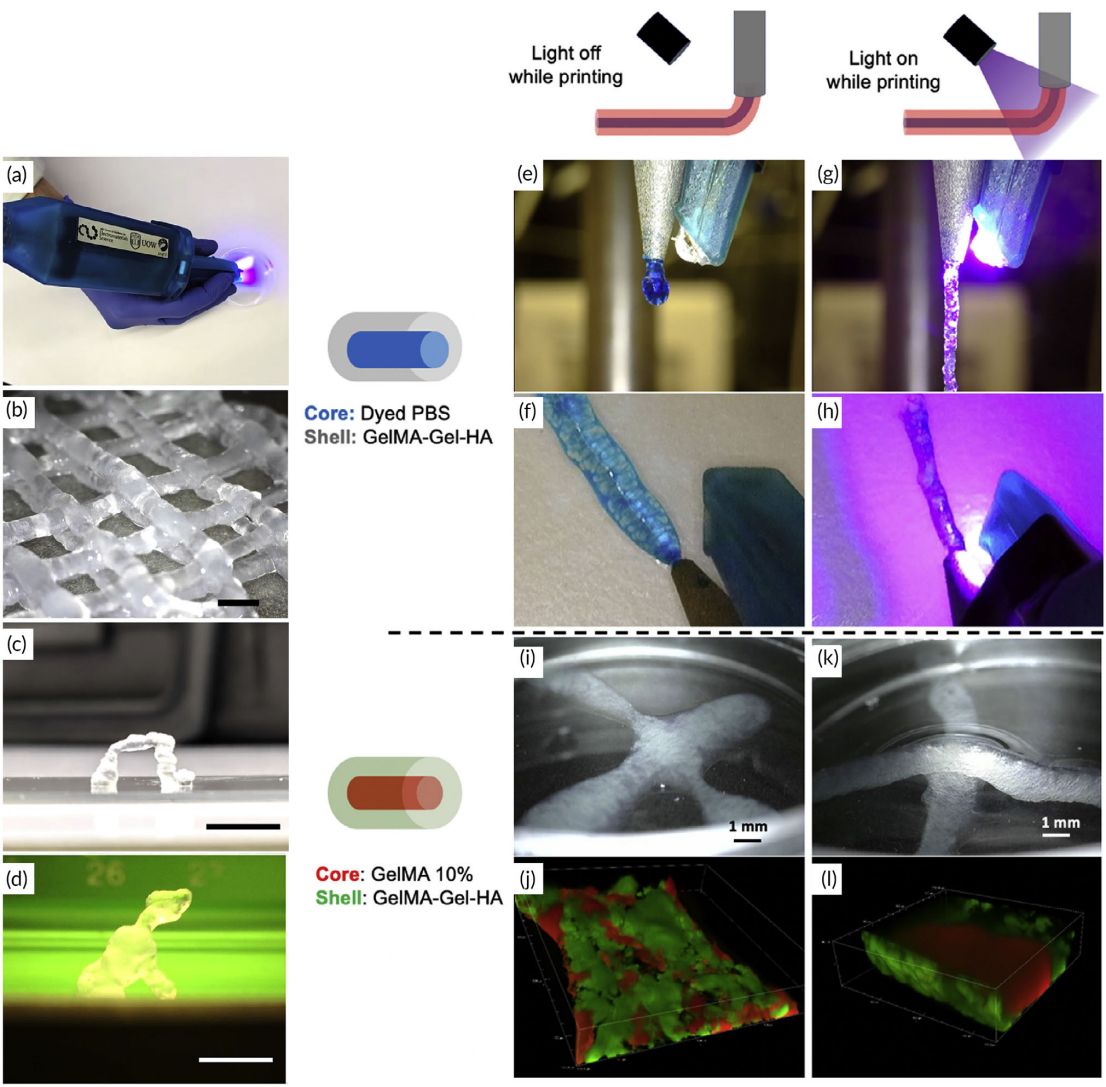
3D sculpting with a hand‐held device. (a) A photograph of our portable extrusion equipment. (b) Log‐pile structure printed by hand. (c) A free‐standing arch that was printed by hand. (d) A hand‐printed miniature sculpture of a dog, measuring roughly 10 mm in height and 10 mm in width. All structures were printed using the optimized GelMA: HA bio‐ink gel. Moreover, a liquid core may be encapsulated co‐axially via in situ photo‐crosslinking printing: (e,f) co‐axial filaments extruded without light exposure (dyed PBS in the core, GelMA: Gel: HA in the shell). The two components immediately mix together during extrusion to produce a liquid droplet. (g,h) Co‐axial filaments (dyed PBS in the core, GelMA: Gel: HA in the shell) in stereomicrographs with light on during extrusion. The shape of the filament is preserved, and it can be deposited as a continuous thread. (i) Stereo‐micrograph of co‐axial filaments extruded without light exposure and subsequently photo‐crosslinked (GelMA 10% in the core, GelMA: Gel: HA in the shell). Rather than stacking, two crossed filaments blend together. (j) Confocal microscopy shows a line printed without light exposure, demonstrating the mixing of the core (red) and shell (green). (k) Stereo‐micrograph of co‐axial filaments with the light on during extrusion (GelMA 10% in the core, GelMA: Gel: HA in the shell), producing two stacked filaments. (l) Confocal microscopy of a line printed with light on during extrusion, displaying the core (red) and shell (green). *Source*: All pictures were reprinted with permission from reference 46

Biopens are novel tools that have created a new approach in the field of bioprinters to transfer engineering facilities into the field of surgery (Figure 2). Among these devices' advantages is the increased speed of operation and surgical skills, portability, flexibility in in situ printing, and practicality by hand.^60^ The most prominent advantage of hand‐held bioprinters is that the operator can hold the bio‐printer to print bio‐ink directly at the wound site, without needing a computer system and defect scanning. Various natural and synthetic biomaterials have been prepared and used as bio‐ink in portable bio‐printer that have applications in wound dressings to treat acute and chronic wounds, bone and cartilage disease, skeletal muscle injury and dental trauma. Also, gelatin methacryloyl (GelMA) and chitosan methacryloyl hydrogels are biocompatible and biodegradable hydrogels that have recently been widely used in various fields of medical engineering. These hydrogels with a concentration range of 5–20 wt% can be mixed with cells and extruded by an improved printer into an X‐Z robotic system portable bioprinter.^61^

An affordable and versatile tool for creating functional materials at the point‐of‐carry (POC) has been developed by BioPen.^62^ POC TE applications benefit from the technology's versatility, which allows cells and proteins to be written on a variety of substrates. Inkjet printing of cells and ECM proteins has three major drawbacks that BioPen addresses. To begin with, BioPen allows for a continuous stream of cells and ECM proteins by directly writing, overcoming the complicated synchronization between nozzle movement, the flow of liquid, and the movement of cells. Second, because the BioPen is in direct touch with the substrate, it can produce exact patterns even when the substrate deforms or displaces noticeably as a result of the deposited biomaterial. Third, BioPen is able to write on curved surfaces, which is challenging for current printing methods. BioPen holds particular promise for low‐cost POC diagnostics, which is an urgent requirement.^62^

### 2.3 The ergonomics of the portable bioprinter

In addition to their obvious importance in health care, medical gadgets also play a significant role in the product development. One of the essential design components of medical devices is the product's ergonomics, which have been designed, engineered, and prototyped for over 20 years.^63^ Ergonomics is a branch of applied science that focuses on the design features of an object that humans can use efficiently and safely. No single explanation exists for ergonomics, which is the absolute truth of the matter. Designing to fit a user's needs is what ergonomics is all about.

There may be more than one person who uses a product. Considering this as one of the most significant situations, it is not just the end‐user who benefits from ergonomic product design; it is for everyone. Three nominal factors in ergonomic design include the product's end‐users, building and assembling the product by the end‐user, and the responsibility of users for maintaining and repairing the product.^64^

Multiple users may be involved at various phases of a product's lifecycle. Hospitals are an appropriate example of this, as both patients and nurses may use the same medical equipment in different ways. In the course of using a medical device, whether it is a hospital bed or an MRI table, each individual has distinct requirements. Product flaws occur when users' needs are met at one stage but not at another. During the conceptual stages of product development, ergonomic design concepts begin to be integrated into a fully ergonomic product. Attention to the user's needs is often overlooked and not evaluated.

In designing devices, all users should be considered. For example, individual users who interact with a portable bioprinter all touch it differently. The user is strongly influenced by the design of the tool, especially when the fingertips and the tool meet. The diversity of human hands is a major challenge in handicrafts design. An outstanding device should achieve the highest level of usability among a population of different users. The vital point in designing portable bioprinters is to find the optimal point of dimensions in which the tool is perfectly proportioned to the size of the hand and provides maximum accuracy and skillful control for the largest possible number of users.

The ergonomics of the device should be designed to be easily held by the user. The structure of the device should have a protrusion near the palm and a recess near the fingertip area for easy handling. The system should also allow the user to control the extrusion of the material. This process is done by installing a start and stop button. During the extrusion process, the curing lamp is set to a low‐power setting to allow for a precure that may be tailored to any pattern or percentage of full power. Intermittent use of the curing lamp during extrusion can be used to impart desired mechanical qualities to the extruded product.

Hand‐held and hand‐intensive products are fundamentally influenced by the hand's inherent geometry. Three palmar arches, known as the grip axis, appear when a user shuts his or her fingers and grasps an instrument. The location of fingertip grip surfaces and controls on a device should be defined in terms of precision, control, and comfort based on hand geometry and size and strength.

Hands are extremely sensitive to touch that a raised dot on a piece of glass that is only 3 mm high may be detected by a fingertip, but a computer mouse with a height of 0.009 in. can be detected by a palm. The combination of the product's tactile sensitivity and physical features defines its haptic signature. The dexterity, precision, control, and comfort of a product are all enhanced when the controls are placed where the user's fingertips naturally land on the device's surfaces and are the ideal size for the user's range of hands.

Form, balance, scale, weight, textures, and materials all contribute to a product's haptic signature, which defines its hand feel. Balancing is an essential consideration in designing most portable or hand‐intensive devices. Upon designing an instrument, keeping the instrument's center of gravity as close to the precision grip's virtual center point is preferred as possible. Precision and control can be improved by reducing the movement of the arms and pendulum effect.

Haptic design decisions are made more complex with tethered handpieces. For example, cable whiplash affects how the gadget feels when held in your hand. Power, suction, and irrigation lines (usually located at the device's rear) confound haptics and balance even further. As a result of all these circumstances, the device's tip loses precision and control and must be regularly counterbalanced by the user. To reduce the line tug on the handpiece's back, using free‐floating articulating strain reliefs is recommended whenever possible in the design.

Fingers on the human hand are both smart and dumb. When it comes to high‐precision and high‐control jobs using hand‐held gadgets, the dominant hand's thumb, index, and middle fingers are the most dexterous. This three‐finger grip maximizes the usefulness of mobile devices' smart fingers. When a device or equipment is being rotated or twiddled by surgeons and doctors, these are the fingers used for maximum precision and control. As a rule of thumb, control surfaces should be sculpted to allow the user's fingertip control to steer and manipulate the instrument, as per ergonomics. Each of the three fingers of the trilateral grip should have a corresponding indent on these types of control surfaces. This will help players with their fine motor skills and speed.

Right‐handed and left‐handed users alike should be able to operate the same ergonomic control surfaces with equal ease. Textured surfaces are common on ergonomic control surfaces because they help grip and control.^65^ It is pivotal to know what kind of traction the gadget needs before designing a texture that would allow the user's fingers to bite in and give that traction. Hand‐held medical equipment's control surfaces are heavily influenced by the ergonomic design idea of dynamic grip security.^66^ Hand‐held gadgets with dynamic grip security allow users to safely grasp an instrument while using fingertip controls on the same hand. This action is usually required in medical equipment that requires the user to hold it. Creating product forms that allow the user to apply a combination of precision and power grips is a challenge for the designers. Using an ergonomic approach based on the natural hand geometry, the ring and baby fingers can be combined into one powerful super finger. Because of this, the thumb, index, and middle fingers may be used in a more dexterous and precise trilateral precision grip in conjunction with the control surface that is specifically created for them.^66^

For portable medical devices, ergonomic design is not limited to these five factors: user focus, hand architecture/dynamics, attention to detail, complicated grip solutions, and tactile control surfaces. No hand‐held or hand‐intensive device can function properly without them, though. In order to create the best devices, ergonomics is at the center of the design process.

### 2.4 Considerations for cross‐linking of printed bioinks

Bioinks used in extrusion‐based printing must maintain the survival, motility, and proper metabolism of the cells integrated into the extrusion‐based printing process. Printing sturdy, thick, multilayer constructions at high resolution typically necessitates additional processing due to the fragile and delicate nature of the materials that meet these characteristics. However, it is possible to print more substantial materials with greater mechanical endurance that can cross‐link at longer intervals, provided that the cross‐linker can permeate the printed material efficiently. Depending on the printing procedure, most bioinks require a stabilizing cross‐linking step before or after printing. The molecular structure of the printed material should be strengthened in order to keep the extruded bioink's form, avoid collapsing, and build firm structures that can withstand the conditions of in vitro culture and/or implantation. Incorporating cross‐links between the polymer molecules is one way to do this, which results in a material that is both self‐supported and noticeably stiffer. In order to maintain the bioink's cytocompatibility, this change must be light enough.

**FIGURE 3:**
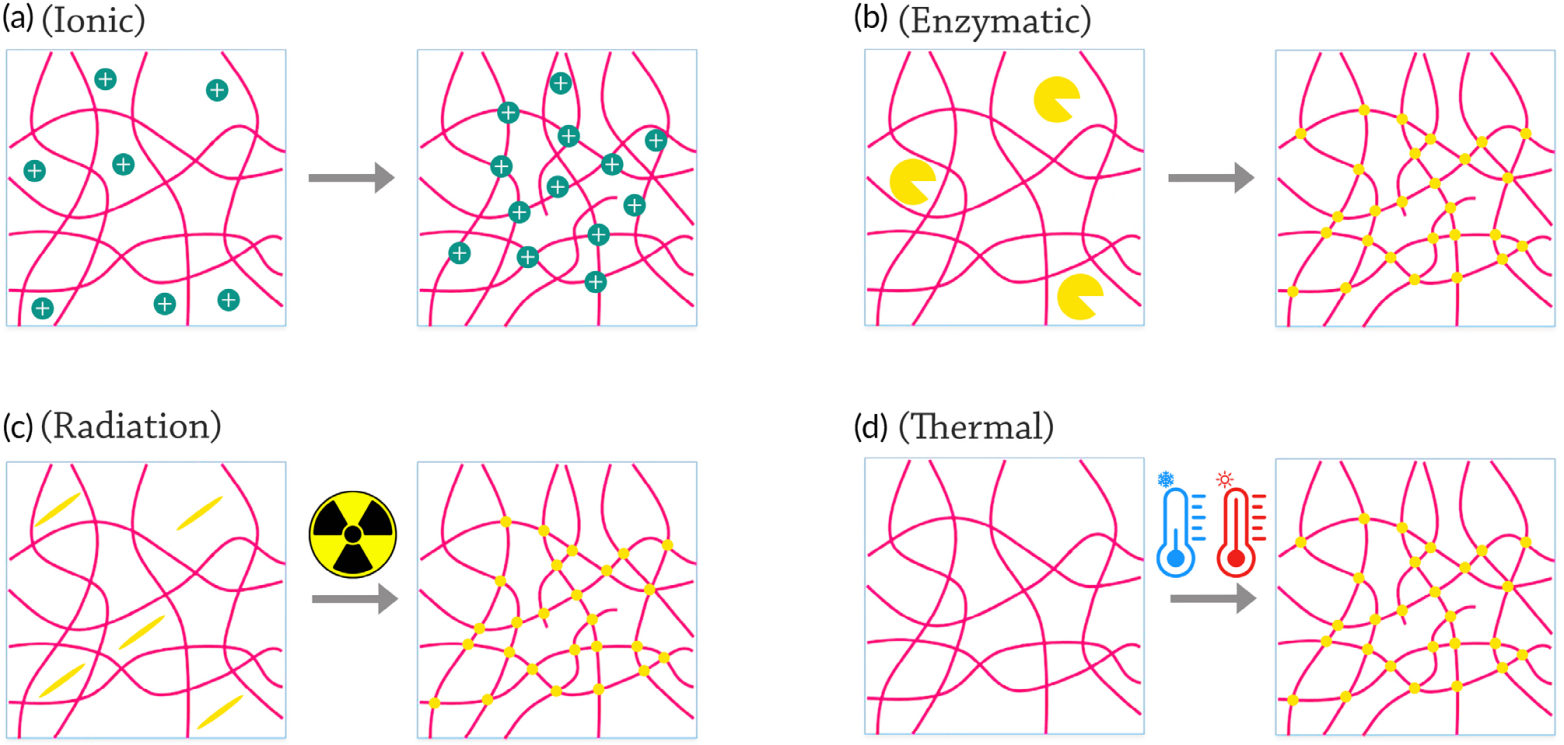
Various bioink cross‐linking strategies that could be used in bioprinting through portable hand‐held bioprinters. (a) Ionic cross‐linking: A simplified schematic depicting the formation of electrostatic bonds among ions (green) and polymer chains with opposite charges, resulting in a branched network. (b) Enzymatic cross‐linking: A simplified schematic depicting the enzyme‐catalyzed formation of covalent bonds among polymeric biomolecules. (c) Light‐induced cross‐linking: A simplified representation of the cross‐linking process in the presence of a photo initiator (yellow). When exposed to UV light, the photo initiator produces free radicals, which start a polymerization process between the functionalized chains. (d) Thermal energy‐induced cross‐linking: A simplified diagram depicting the structural changes that occur as a result of temperature variations, resulting in the formation of polymer chains

A bioink's mechanical qualities, its ability to retain its form after exiting a nozzle, and the composition of a cross‐linker all influence the timing of the crosslinking process.^3^ When pressure is exerted from the upper layers, weak hydrogels tend to spread over the substrate and distort. As a result, when using hydrogels, cross‐linking must be done simultaneously as extrusion or before adding a fresh layer of bioink. This also applies to cross‐linking agents that cannot penetrate deep into multi‐layered structures. There are several ways that the cross‐linker can be introduced, such as by coaxial extrusion of the bioink; spraying, deposition, or projection of the cross‐linker onto new layers; or printing into a crosslinking‐inciting environment.^3^ The most prevalent methods for reinforcing printed bioinks are based on ionic, enzymatic, light irradiation, and thermal energy‐induced cross‐linking (Figure 3).^67^

### 2.5 Effective factors on the survival of printed cells

The printing process relies on a thorough knowledge of the effects of shear stress, compression, cavitation, and other external stresses, such as those induced by 3D printing systems, on cell survival and proliferation. For cell carriers, shear‐thinning behavior, that viscosity decreased by shear, and immediate gelation properties are ideal, while extrusion of biomaterials that present shear‐thickening, that viscosity increased by shear, characteristics along with unfavorable gelling mechanisms affects cell viability, resulting in cell death and injury.^68^

A bioprinter's ability to maintain viable cells is critical. Multiple factors impact cell survival during bioprinting, such as cell compatibility and rheological qualities, as well as cross‐linking techniques.^69^ Extruded nozzle shear stress is inevitable when the bioink is loaded with cells. Therefore, it plays a critical role in determining cell viability during and after bioprinting. The degree of shear stress given to the encapsulated cells is principally affected by the nozzle diameter, bioprinting pressure, viscosity, and shear‐thinning specificity. Cell signaling has long been influenced by shear stress. As shear stress increases intracellular calcium levels, downstream signaling pathways are affected. Changes in reproduction and differentiation can result from these factors. When the cell membrane ruptures due to excessive shear pressures, it can lead to cell death. Pressure, extrusion technique, nozzle diameter, printing temperature, and polymer concentration all play pivotal roles in portable extrusion bioprinters. It is possible to print with both conical and straight nozzles. There are nonshear tensile stresses that emerge from the contraction of a syringe at the nozzle in both structures. During this process, cells deform but do not rotate around a central point, which dramatically leads to cell death. In multiple studies, direct nozzles have been demonstrated to have much worse cell survival than conical nozzles.

**FIGURE 4:**
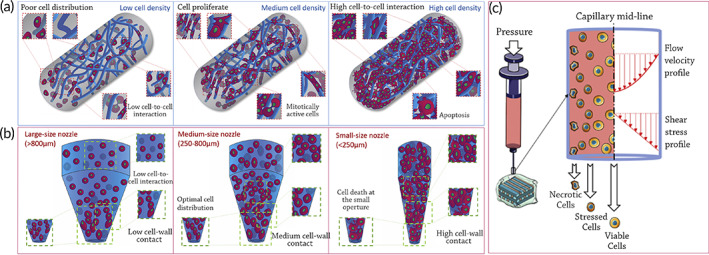
The effect of cell seeding density and shear rate on 3D cell‐laden biomaterial strands and nozzle extrusion. (a) Extruded cell‐laden filaments may contain a number of cells proportionate to the density of cell seeding. The concentration and distribution of polymeric chains (dark blue) have a direct impact on cell proliferation potential. Poor cell seeding density causes poor cell dispersion within the printed strand, resulting in low cell‐to‐cell contact and a restricted growth rate. Physical pressures exerted by neighboring cells restrict proliferation and survival. Cell density may be adjusted to provide a uniform distribution of cells enclosed inside the printed filament, allowing cells to retain the necessary contact with other cells in order to stay mitotically active and grow. (b) Keeping the number of cells in a printing syringe constant but altering the nozzle aperture will impact cell printability. Large nozzles (>800 m) provide limited cell‐nozzle barriers and cell‐to‐cell contacts, resulting in a wider dispersion of suspended cells inside the bioink. These parameters may assure good cell survival after extrusion but result in poor overall construct resolution. Narrower nozzles (250 m) with a lower surface area for the same amount of cells, on the other hand, compel paste encapsulated cells to contact with one another, resulting in high density at the nozzle aperture. Upon printing, a tiny orifice may generate excellent resolution as well as significant cell death. Medium size conical nozzles (250–800 m) provide excellent cell dispersion inside the nozzle and an improvement in print resolution without affecting cell survival significantly; *Source*: reproduced with permission from reference 69 (c) Shear stress's effect on cell viability during extrusion‐based bioprinting (the system used in portable hand‐held bioprinters). Cells along the wall are exposed to greater shear stress intensity than cells in the middle of the nozzle, resulting in damage. Cell viability diminishes exponentially as shear stress rises

In addition to the nozzle design, the diameter of the distribution orifice also impacts cell survival.^70^ Figure 4 illustrates the impact of the nozzle design and diameter on the cell's interactions and survival. Shear stresses grow with increasing viscosity or storage modulus, which requires more enormous pressures to extrude it.

In cell printing, the shear field at the nozzle, which is considered the principal source of cell damage and loss, is of particular importance. Through shear, mechanical cell disruption occurs during bioink extrusion from syringe nozzles. Fluid near the nozzle walls is thinned while still flowing laminarly. Cells are immediately exposed to a velocity gradient profile that varies depending on the nozzle used throughout the printing process, typically a maximum intensity at the center of the nozzle, leaving a static field around the nozzle wall.^71^

Shear‐thinning and nonshear‐thinning biomaterials are employed in bioinks. During the application of shear force, shear‐thinning materials can be injected and have the potential to swiftly self‐heal. When physical or chemical stimuli are applied to nonshear thinning materials, gelation occurs. In extrusion, shear stress is maximized at the nozzle walls. Equation (2) shows the relation among the shear stress (*τ*_wall_) and the distance along the nozzle radius (*R*).^72^ Also, ∆P presents the pressure drop in the cylindrical nozzle, while the total length of the cylindrical nozzle is showed by L.

(2) $$\;\tau_{\text{wall}}=∆P.\frac{R}{2L}$$

As a result, printing settings must be adjusted in accordance with the intended bioink. Bioinks may be printed accurately using blunt cylindrical nozzles, both with and without cells. Given the strong shear stress field generated at the contact of the nozzle during printing, the cylindrical nozzle design may compromise and harm cells. According to recent studies in which cell‐compliant conical nozzles have replaced cylindrical nozzles, shear stress and polymer concentration studied cell survival following extrusion in both nozzle types.^69^ Conical rather than cylindrical nozzles appear to have less of an effect on cells. As a result, cell viability after printing with cylindrical nozzles was around 10 times lower than with conical nozzles of the same gauge.^73^

Modeling the 3D bioprinting process and the impact of bioprinting parameters on cell viability is a useful tool that may be used to establish ideal experimental conditions. Most cell damage laws are based on a power‐law function (Equation 3), which simply links the percentage of cell damage to shear stress, as shear stress is thought to be a major influencer on cell damage.^74^ In this equation, *y*· represents the shear rate, *K* shows the consistency index of the hydrogel related to viscosity, and n is a power‐law exponent.

(3) $$\tau_{\text{wall}=}\;{\mathrm{Ky}}^{n}$$

Furthermore, shear, tensile, and compression stresses, in addition to the forces created by matrix topography and stiffness, have been shown to lead to stem cell differentiation.^75^ Cellular activities were shown to be greatly affected by shear stress.^76^ Shear stress is an essential microenvironment element for controlling the growth of stem cells. The mechano‐sensitive cation channels in stem cells, like those in other types of cells, translate mechanical inputs into biochemical and biological reactions.^77^ Protein conformational changes, such as those caused by hemodynamic flow in the body or urine, might lead to the exposure of functional domains or binding sites.

Stem cell differentiation is influenced by shear stress, including pulsatile, oscillatory and turbulent flows. Specifically, shear stress directs stem cell development toward endothelial and bone‐producing cell types that encounter significant mechanical stresses in vivo. Increased ATPase activity and calcium deposits were seen after stem cells were subjected to higher shear stress levels.^74^ Overall, two key elements, material qualities and printing settings govern the effect of process‐induced shear stress in extrusion‐based bioprinting.^75^

## 3 TISSUE REGENERATION VIA PORTABLE BIOPRINTERS

A variety of biomaterials were printed through utilizing hand‐held portable bioprinters in skin, skeletal muscle, cartilage, bone, and dental regeneration. Table 1 summarizes all the tissues, bioink materials, crosslinking materials, drugs, cells, models, and bioprinters used in several studies. Although it has not covered all the body tissues, it possesses the high potential of spreading to other tissues.

**TABLE 1:** Summary of tissue types have been prepared via portable and in situ bioprinting

Type of tissue		Biomaterial/bioink	Crosslink material	Drug or growth factor	Cell type	Cell source	Bio‐printer type	In vivo animal model	References
Soft Tissue	Skin	Fibrinogen, HA, collagen, alginate	Calcium chloride and thrombin	Tramadol	Keratinocyte	Allogeneic or autologous	Portable hand‐held extrusion	Porcine wounds	53
		Fibrin based and collagen	Thrombin solution	‐	Mesenchymal stem cells (MSCs)	‐	Hand‐held bioprinter	Porcine full‐thickness burn	78
		Fibrinogen and collagen	‐	‐	Fibroblasts and keratinocytes	Autologous or allogeneic	In situ bioprinting	Mice and porcine models	79
		GelMA and PEO	Irgacure2959	‐	NIH/3 T3 and HUVEC	‐	Hand‐held bioprinter	‐	51
	Skeletal Muscle	GelMA	Irgacure2959	‐	Muscle cells	‐	Hand‐held s 3D bioprinters are extrusion	Mice with volumetric muscle loss (VML) injury	80
	Cartilage	GelMA, gelatin, HA	VA‐086 pho‐toinitator	‐	Human adipose stem cells (hASCs)	Human infrapatellar fat pad	Hand‐held pneumatic extrusion	‐	60
		GelMA and Host‐Guest Macromer (HGM)	Irgacure2959	Kartogenin and proteinaceous (TGF‐β1)	Human bone marrow‐derived mesenchymal stem cells (hBMSCs)	‐	In situ injection	‐	81
		GelMA and HAMa	Irgacure2959 and lithium‐acylphosphinate (LAP)	‐	Sheep adipose‐derived stromal/stem cells (ADSCs)	Sheep infrapatellar fat pad (IPFP)	Coaxial bioprinter	‐	82
		GelMA and HAMa	Irgacure2959	‐	Human‐derived mesenchymal stem cells (hADSCs)	Infra‐patellar fat pad of donor patients	In situ bioprinting (co‐axial extrusion Biopen)	Sheep model	83
		HAMa	Acrylate‐terminated four‐armed polyethylene glycol (4‐armed PEG‐ACLT)	‐	MSCs	‐	Six‐degree‐of freedom (6‐DOF) robot in situ bioprinter	Rabbit	84
		(HA‐GelMA) hydrogel	Irgacure2959	‐	MSCs	‐	Co axial hand‐held bioprinter (Biopen)	Sheep	52
		Sodium hyaluronic acid (HA)‐based hydrogel	Irgacure2959 Cacl2	‐	‐	‐	In situ bioprinting	Sacrificed 4‐month‐old New Zealand rabbit	85
Hard Tissue	Bone	Sodium alginate and polyethylene (glycol) diacrylate (PEGDA)	Irgacure2959 Cacl2	Bone morphogenetic protein 2 (BMP‐2)	‐	‐	In situ bioprinting	Sacrificed 6‐month‐old Bama mini pig	85
		Polycaprolactone (PCL), Zinc oxide nanoparticles and hydroxyapatite (HAp) particles	‐	‐	MSCs	‐	*Pen Bone portable printer*	C57BL/6 mice	86
		Collagen and nano‐HAp	‐	‐	MSCs	‐	Laser‐assisted in situ bioprinting	Calvaria defect model in murine	12
	Dental	Agarose (AG), collagen type I (COL1), and fibrin (FIB)	Tris‐buffered saline, thrombin, calcium chloride	‐	Human primary dental pulp cells and endothelial cells	Extracted third molars of patients,	In situ bioprinting	Human root canals	87

### 3.1 Soft tissue regeneration

#### 3.1.1 Skin regeneration

The biggest organ in the body, the skin is composed of extracellular matrix (ECM) and a unique layered arrangement of cells.^88^, ^89^ The epidermis, dermis, and hypodermis are the three layers of the skin. The viable epidermis is the outermost cellular layer that is densely packed with keratinocytes and acts as a barrier against dehydration and bacterial penetration. The dermis layer lies under the epidermis and it is occupied by fibroblasts and a variety of other cell types, as well as a thick ECM containing collagen. The hypodermis is the last layer, which includes adipose lobules and a variety of skin appendages such as blood vessels, hair follicles, and sensory neurons.^90^

Patients with chronic and acute full‐thickness wounds are particularly prone to opportunistic dehydration and infection. Full‐thickness wounds, in which the epidermis, dermis, and hypodermis are damaged, do not heal fully or take a long time to cure due to the reconstruction of the dermis followed by re‐epithelialization moving from the wound edge.^91^ The main therapies for wound closure and protection are critical stages in increasing patients' life by preventing wounds from worsening for an extended period of time, causing further tissue damage and resulting in long‐term hypertrophic scarring.^79^

Skin autografts are widely regarded as the gold standard for wound healing.^92^ Due to the limited amount of good donor skin, wound dressing is frequently complex and challenging and allografts also have a significant risk of immunological failure of the skin graft.^93^ TE approaches in constructing biologically engineered skin are an appropriate alternative to skin autografts.^94^ After assessing effectiveness in healing and regenerating burn or chronic skin wounds, the bioprinting skin cells, keratinocytes and fibroblasts layer, and biomaterials have been studied for translational study.^95^

Skin replacements in a variety of shapes and sizes and dimensions are similarly difficult to produce. As a result, covering skin imperfections with varying depths or surface features is challenging and in situ or portable bioprinting of skin cells using appropriate biomaterials has demonstrated encouraging results.^39^, ^72^ Bioprinting techniques for constructing skin tissues outside the body have demonstrated improved wound dressing and skin regeneration in vitro and in vivo.^73^ Although a few research testing handheld and portable skin bioprinters have been conducted.

**FIGURE 5:**
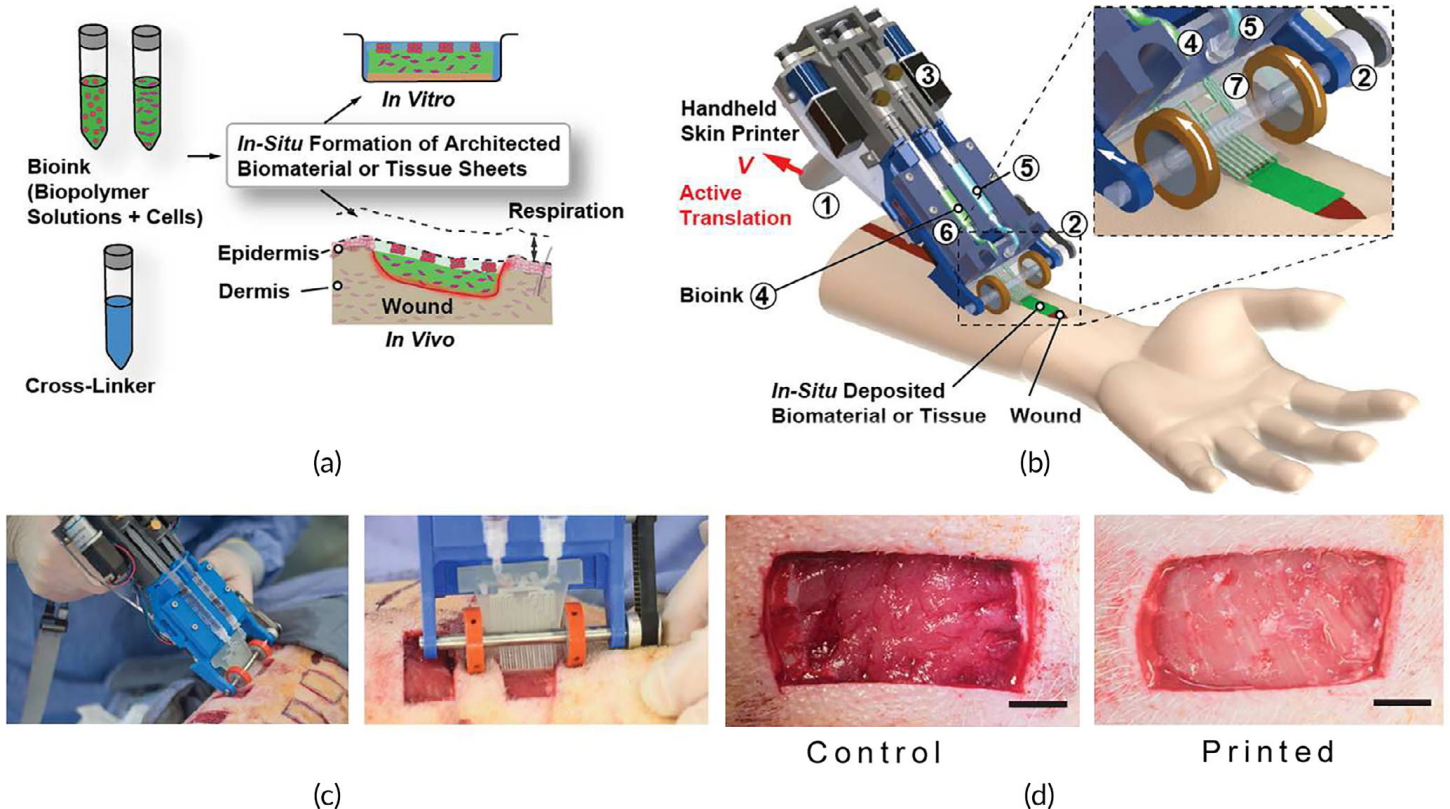
Skin printer on a hand‐held device (a) A schematic illustration depicting the operation of a hand‐held bioprinter. A cross‐linker solution (blue color) and one or more bio‐ink solutions (green color) comprising premixed biomaterials and cells are produced. In a culture dish or at a wound site, a hand‐held bioprinter transforms bio‐inks into homogeneous or architected biomaterial sheets or tissues. (b) Hand‐held bioprinter rendered picture. A handle (1) allows you to place yourself above the target surface or wound. The deposition speed, V, is determined by a stepper motor, pulley, and driving mechanism (2). The dispensing flow rates for bioink (4) and cross‐linker solution are controlled by two on‐board syringe pump modules (3). (5). For spatial organization of solutions and sheet production, a 3D manufactured microfluidic cartridge (6) was used. (c) Representative image showing in situ deposition of a fibrin‐HA/collagen sheet on top of a full thickness excisional porcine wound using a hand‐held skin printer (left); close‐up view of sheet formation in wound bed with a w0 = 2 cm microfluidic cartridge (right); (d) (control, not printed) on Day 0 and Printed 5 min after in situ formation of biomaterial sheet. *Source*: All images were reprinted with permission from reference 53

Hakimi et al.^53^ created a new sort of hand‐held extrusion‐based skin bioprinter (weight 0.8 kg) capable of depositing tissue sheets or biomaterials from a microfluidic cartridge. This design enables in situ bioprinting of minor or big animal skin wounds. The stepper motor, through pulley and driving mechanisms, and two syringe pump modules may be used to adjust deposition speed and bio‐ink supply or flow rates. The basic notion of portable bioprinting is depicted in Figure 5a,b. Suspended cells (allogeneic or autologous) are put into one or multiple independent syringes using the hydrogel precursor solution. Another syringe contains a crosslinking solution that aids in the gelation of the cell‐laden biopolymer solution at moderate circumstances (physiological temperature and pH) and with high cell survival. After loading the syringes into the portable bioprinter, the bio‐ink is put on the culture plate as a biomaterial or tissue sheet for in vitro research or directly into a wound for in vivo studies.

They prepared four kinds of bioinks that were cross‐linked in two ways, chemical and enzymatic. Their bioink included alginate‐collagen sheets, along with fibrin‐based sheets (fibrinogen, HA, collagen), which were cross‐linked via calcium chloride and thrombin to form dermal and epidermal layer, respectively. In their study, the bio‐ink, including keratinocyte cells can be deposited with parallel stripe patterns separated with cell‐free stripes, like a meshed epithelial skin graft.

The authors depicted that porcine wound were covered by prepared homogeneous fibrin‐sodium hyaluronate sheets in 20 days (Figure 5c,d). Also, they could introduce a wound model in a proof‐of‐principle of in situ bioprinting of four various bioink with tramadol (4‐6 mg/kg) drug to control pain and cells. Their results investigate that in situ bioprinting improves adhesion between dermal and epidermal layers, decreasing scar formation, improving cell differentiation, proliferation, and migration, and influencing tissue formation.^53^

**FIGURE 6:**
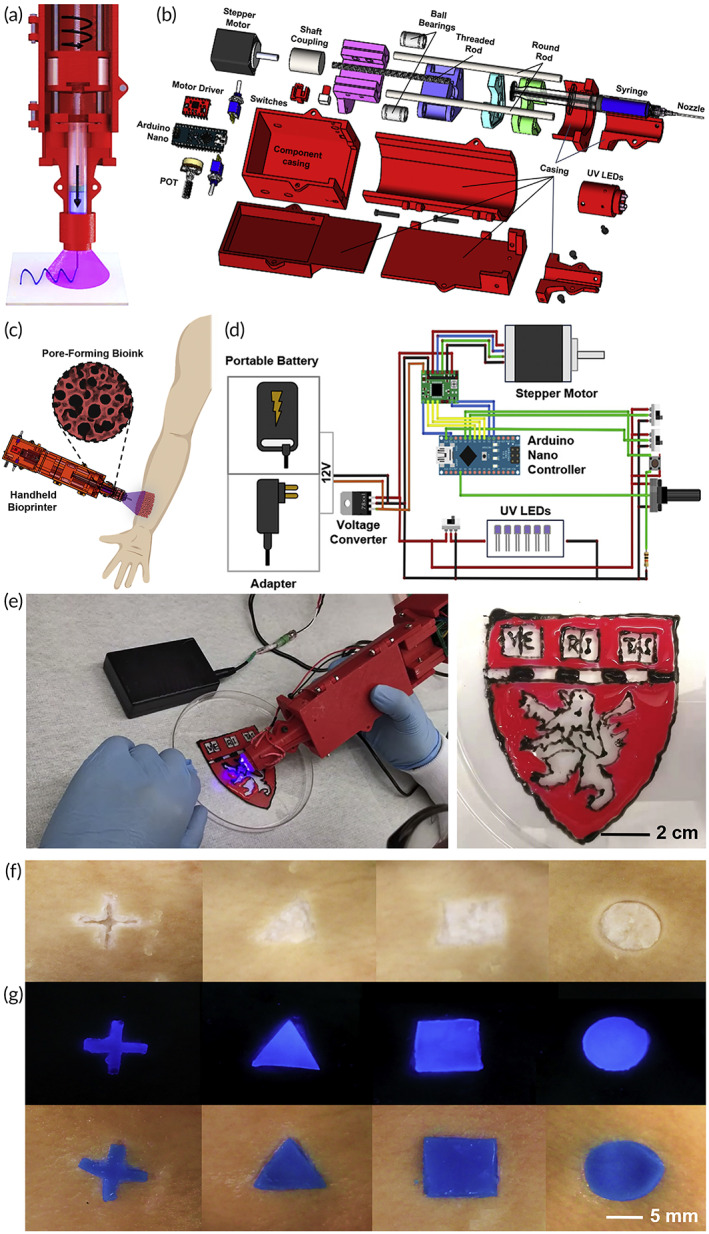
A developed low‐cost hand‐held bioprinter and a unique bioink: (a) A schematic of the hand‐held bioprinter's design. (b) An exploded schematic depiction of the hand‐held bioprinter's components and assembly. (c) Schematic diagram demonstrating the notion of combining a pore‐forming bioink formulation with a low‐cost, open‐source, and ergonomic hand‐held bioprinter for in situ wound dressing. (d) A schematic depicting the design of the hand‐held bioprinter's controlling system. (e) Using pore‐forming bioink colored in various hues, hand‐held bioprinting of a Harvard logo. (f, g) Pore‐forming hydrogel patterns hand‐printed on ex vivo pig skins with artificial wound forms. *Source*: All pictures were reprinted with permission from reference 40

Ying et al.^51^ developed a low‐cost hand‐held bioprinter and unique bioink (Figure 6a–d). The continuous phase was made up of a two‐phase aqueous photo‐cross linkable biopolymer (GelMA), while the emulsion droplets solution was polyethylene oxide (PEO). Pore‐forming bioink was used to immobilize fibroblasts from the NIH/3T3 cell line and human umbilical vein endothelial cells (HUVEC) before printing. They demonstrated that the hand‐held bioprinter was suitable and adequate for clinical applications (Figure 6e–g). Also, they formulated a unique two‐phase aqueous emulsion bioink that provided a perfect environment for cell survival and suitable for wound healing.

**FIGURE 7:**
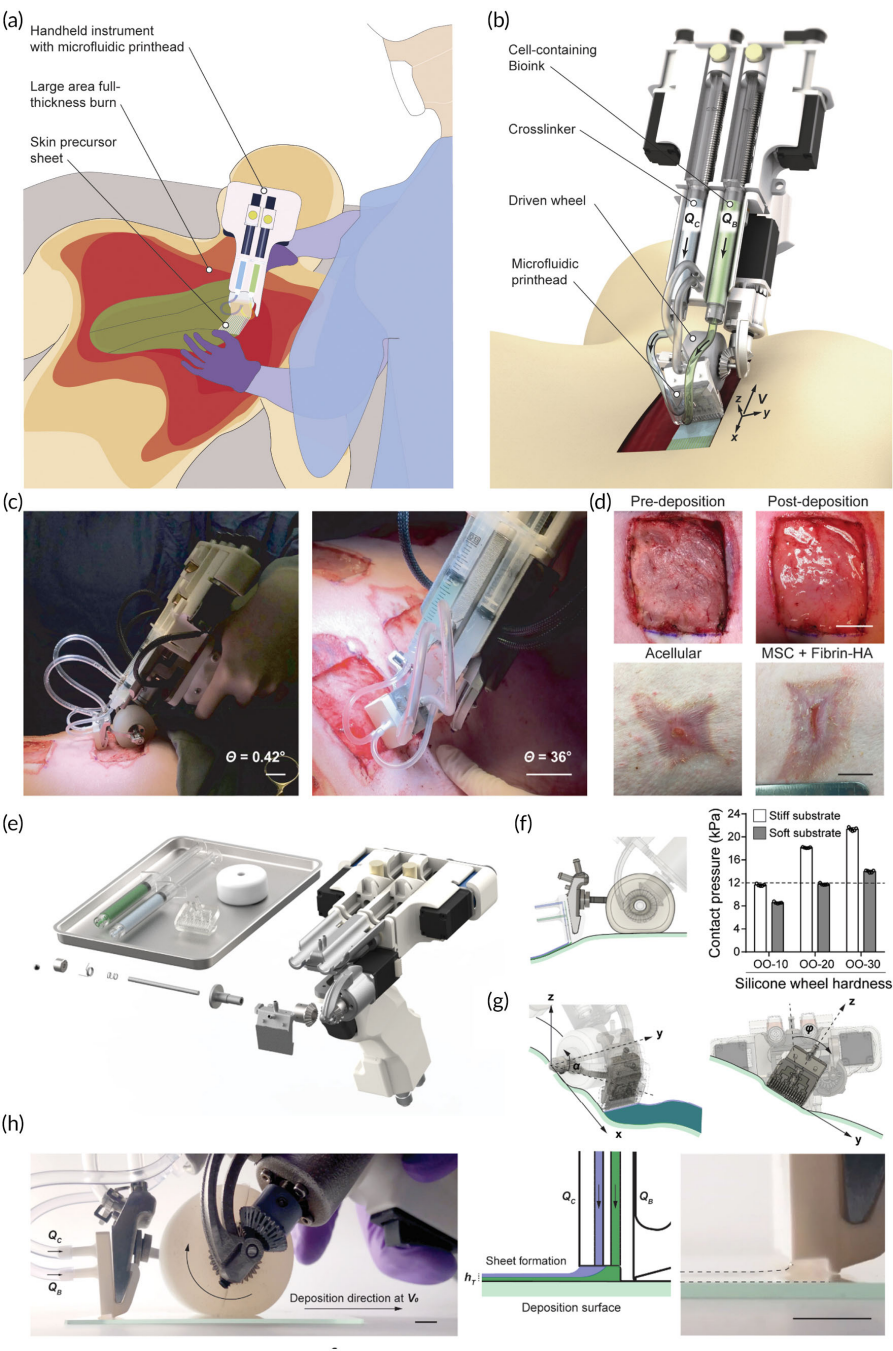
In situ creation of precursor skin tissue. (a) A hand‐held method for putting cell‐laden biomaterial sheets conformal to a full‐thickness burn lesion is shown schematically. (b) Picture of portable device for controlled delivery of bioink consisting of MSCs in fibrin bioink (green color) provided at flow rate (QB) and cross‐linker (clear) supplied at flow rate (QC) through microfluidic print head, while pushed by soft wheel along skin surface at velocity (V). (c, d) MSC‐containing fibrin‐HA biomaterials applied homogeneously on a porcine full‐thickness burn surface with a hand‐held device aid wound healing. 3D rendering, exploded view of hand‐held instrument and disposable bioink syringes, microfluidic printhead, and silicone wheel. Conformal deposition of biomaterial layers onto physiologically relevant topologies (e), 3D rendering, exploded view of hand‐held instrument and disposable bioink syringes, microfluidic printhead, and silicone wheel. (f), Left: Printhead side view demonstrating conformal sheet deposition by printhead onto wound substrate that is unaffected by wheel deformation. Right: contact pressures measured on stiff and soft surfaces for wheels of various hardness. The stiffness of injured tissue is shown by the dotted line. n = 5 separate experiments, data given as mean ± s.d. (g), Left: rendered picture of printhead moving along *y*‐axis with pitch angle, correcting for up to 45° inclinations. Right: printhead rotation around the *x*‐axis with roll angle, compensating for a 25° change in instrument position relative to the deposition surface's normal direction. (h), Photo of bioink extrusion from the side. (QC) and (QB) denote cross‐linker and bioink perfusion through the printhead. The wheel spins clockwise to move the instrument in the deposition direction at nominal speed V0. Middle: a schematic cross‐sectional view of the printhead with bioink and cross‐linker leaving to produce biomaterial sheets on the deposition surface with height (hT). Photo showing fibrin sheet formation on the right. 2 mm scale bar. *Source*: All pictures were reprinted with permission from reference 53

Cheng et al.^78^ designed and fabricated a hand‐held bioprinter, possessing a weight of 1.4 kg, based on microfluidic technique to promote rapid recovery in burns through cell delivery and for real‐time sheet deposition directly onto wound area in an appropriate shape, size, and topography (Figure 7a,b). They used a fibrin‐based bioink, along with a thrombin solution as a cross‐linker. The bioprinter's soft driven wheel can make touch with the wound's surface when the user places the bioprinter on the handle (Figure 7c,d). Each syringe contains bioink and crosslinker, which are administered simultaneously at different flow rates (Figure 7e–h).

This microfluidic print‐head‐based hand‐held device deposits skin sheets straight from cells and biomaterials. Cell survival and proliferation are preserved while using this strategy. The portable instrument's modular architecture makes it easy to operate and adjust the skin sheet's physical dimensions and biomaterial composition. The authors indicated that the hand‐held bioprinter could safely and reliably deposit mesenchymal stem cells (MSCs) containing fibrin‐based skin sheets onto a burning bed to improve wound healing results.^78^ Their study suggest that cell‐containing biomaterial sheets are deposited on the wound like a paint roller, covering the surface with a uniform sheet of skin, stripe by stripe, as well as in vivo trials on full‐thickness wounds.^78^ With the advent of next‐generation safe cell and biomaterial delivery made possible by this automated technology, therapeutic applications that go beyond full‐thickness burn injuries will arise in the future.

**FIGURE 8:**
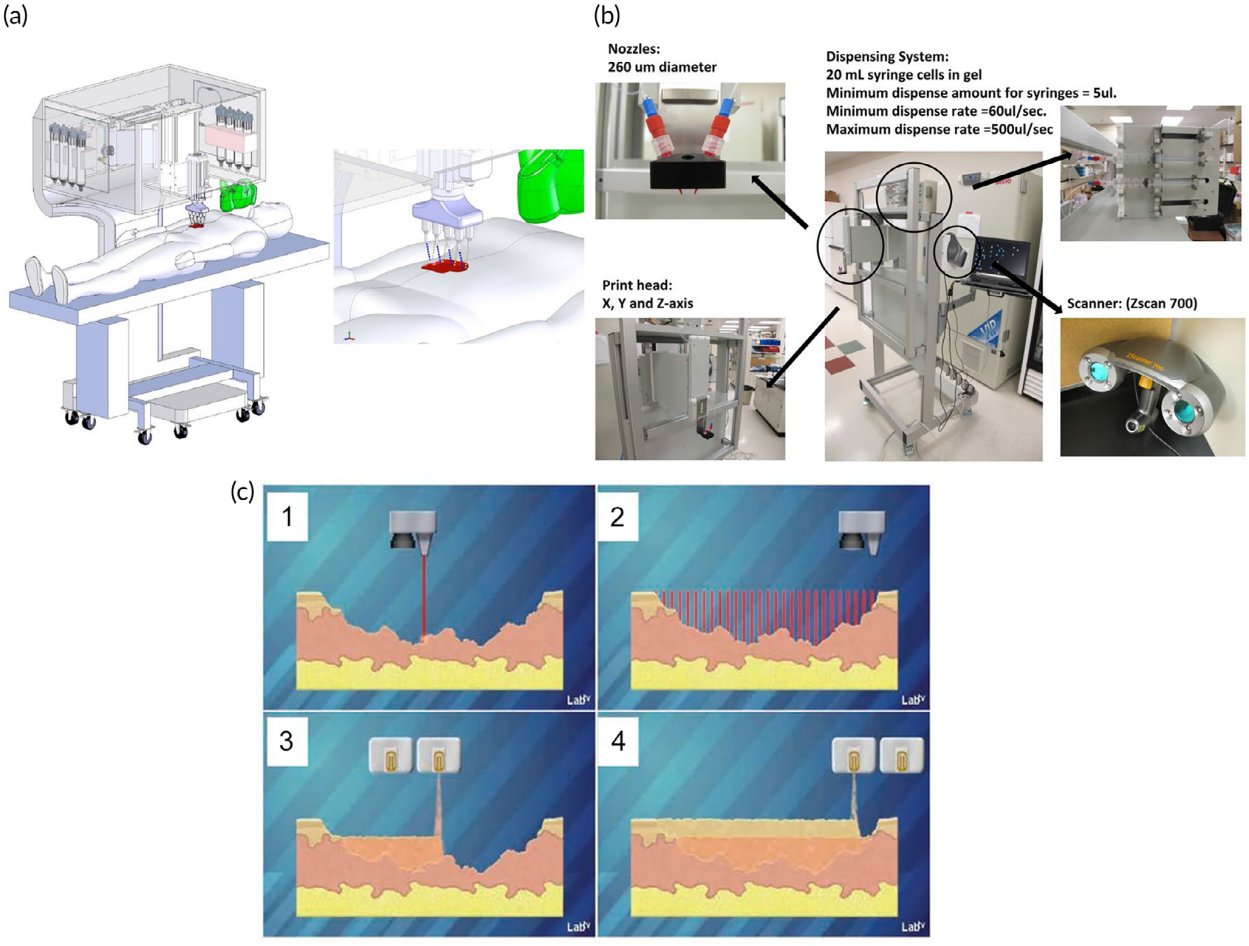
Prototype of a skin bioprinter and idea of in situ bioprinting (a) A schematic of the skin bioprinter's size, design, and components. (b) In addition to the 3D wound scanner, the primary components of the system include 260 m diameter nozzles operated by up to 8 independently dispensing systems coupled to a print‐head with an XYZ movement system. All of the components are installed on a compact frame that may be moved about the operating area. (c) The idea of skin bioprinting. Wounds are scanned first to get accurate information on wound topography, which is then used to direct printheads to deposit specific materials and cell types in the correct areas. *Source*: All pictures were reprinted with permission from reference 79

Recently, Albanna et al.^50^ presented a new in situ skin bioprinting system's design and proof‐of‐concept validation. This device is capable of combining imaging technologies to determine the topography of wounds with precise cell administration to meet the specific demands of each patient (Figure 8a). The authors claimed that the developed bioprinting system has advantages, including hand‐held and capable of being easily transported, identifying and measuring the wound sizes and topologies, delivering various cell types, easy sterilizing, and a low cost.

Wound scanner to accurately assess wound architecture and to allow accurate distribution of appropriate cell types to particular wound surfaces have been integrated in this in situ bioprinter, which marks a significant breakthrough in customized wound treatment. Portable 3D wound scanner and a print‐head with the XYZ movement system, each driven by an independent dispensing motor, are the primary components of the bioprinter. In order to move around the operation area, everything is mounted on a tiny frame (Figure 8b).

Skin wounds can be reconstructed by combining the wound scanning system with the cartridge‐based delivery system (Figure 8c). An in vivo study by the mice and porcine models demonstrated that this in situ bioprinter provided instant and suitable coverage of wound beds by delivering autologous or allogeneic fibroblasts and keratinocytes with the biological hydrogel, such as fibrinogen and collagen.^79^ Thus, wound therapy with our in situ bioprinting method produced quick and appropriate covering of critical wounds for maintaining homeostasis, re‐epithelialization, and scar prevention.^79^

#### 3.1.2 Skeletal muscle regeneration

**FIGURE 9:**
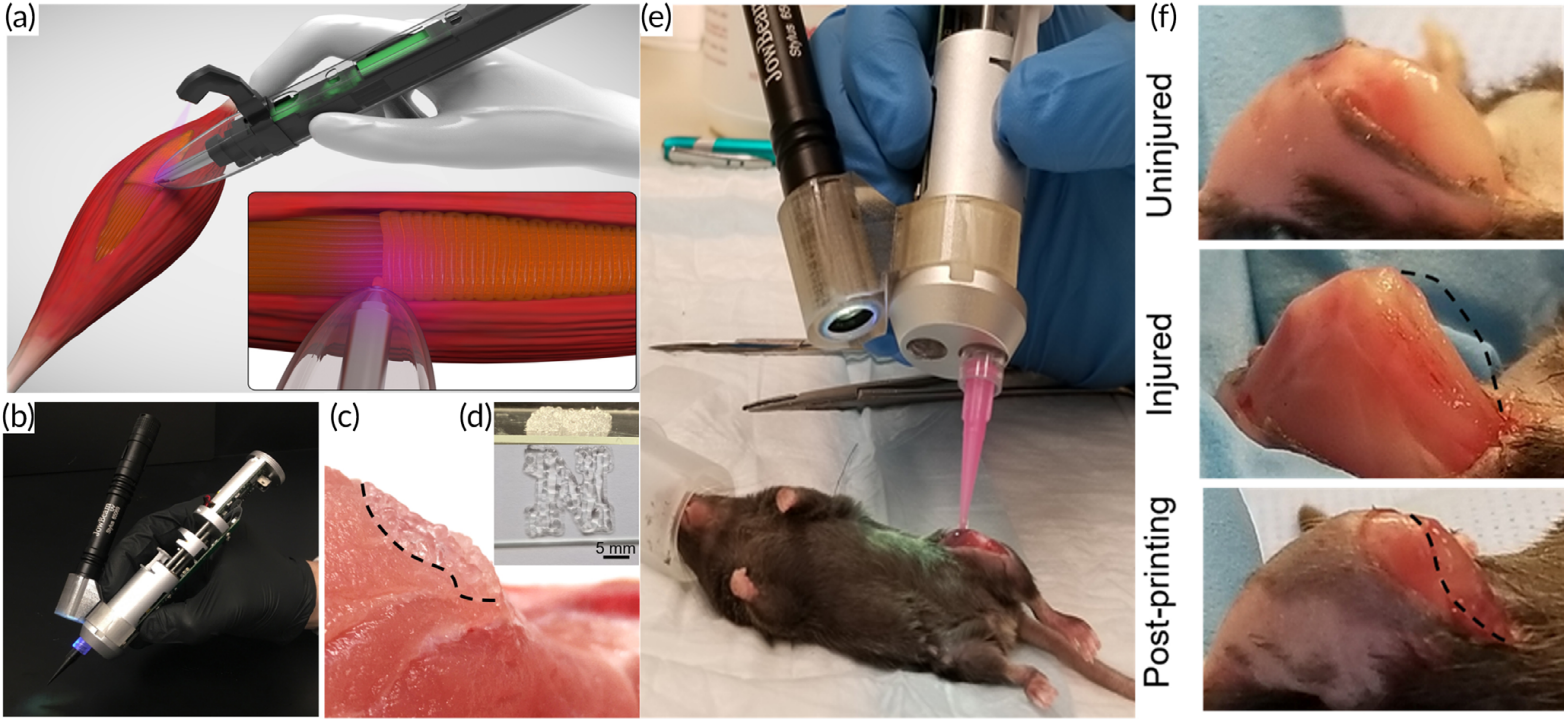
The in situ printing of scaffolds with a hand‐held bioprinter. (a) Schematic of cell‐laden GelMA hydrogels bio‐printed in situ. (b) A photograph of the potable 3D bioprinter that was used, which was equipped with a UV light source for in situ cross‐linking of the printed scaffolds. (c) Illustration of a standard scaffold printed on a nonflat pig skeletal muscle. (d) Photograph of a scaffold in the shape of an N. (e) Surgical implantation of GelMA hydrogels into a murine VML lesion using in situ printing. (f) Before VML surgery, after VML surgery, and after GelMA hydrogel in situ printing. *Source*: All pictures were reprinted with permission from reference 80

Volumetric muscle loss (VML) is caused by skeletal muscle injury as a result of trauma or surgery.^93^ VML is mostly caused by trauma of a severe enough nature to leave victims in a state of social and economic hardship.^94^ Organized and self‐renewing, skeletal muscle is an important part of the human body Because of intrinsic soft tissue damage, a lack of adequate regeneration, and fibrosis, VML injuries have limited ability to recover.^96^ However, conventional reconstructive therapy for such injuries prevents amputation, but they are limited in returning muscle strength and movement.^97^ VML therapy procedures include prosthetic bracing and autogenic muscle flap transplantation, while these treatments have some limitations.^98^ Owing to a lack of definitive therapy, VML leads to constant pain and disability.^99^ The minimal bioavailability of biological growth factors or low cell engraftment has limited the efficiency of regenerative medicine techniques such stem cell and growth factor administration.^93^ Because it produces functioning tissue constructions that can restore damaged muscle function and structure, TE has considerable potential as an alternative therapy.^100^ Portable automated bioprinters capable of printing scaffolds in situ have recently emerged as a potential solution to many of the problems associated with VML damage.^100^ Hydrogel‐based scaffolds were printed into the defect region of mice with VML damage in a study conducted by Russell et al.,^100^ demonstrating adequate adherence to the surrounding tissue and encouraging muscle cell development (Figure 9). Bioprinter allows surgeons to deposit hydrogel‐based scaffold or biomaterial to promote cellular and tissue development into weak regions of skeletal muscles. This technology can be used in the VML therapy procedure, especially when reconstructive surgery has proven insufficient. This hand‐held bioprinter is robust and provides an appropriate filling of the cavity by fibrillar scaffolds in which fibers simulate the muscle tissue architecture. Also, this approach does not need the presence of sophisticated imaging and printing systems. These hand‐held s 3D bioprinters are extrusion based and can be used for bioprinting on targeted directly, overcoming traditionally stationary 3D printers' limitations. The hand‐held bioprinter, a partially automated, extrusion‐based tool, can continuously extrude biomaterials that include the light source for crosslinking the extruded bioink.

Hand‐held, automated bioprinters have the benefit of overcoming some of the disadvantages of static bioprinters, such as the difficulty to print on nonflat surfaces, restricted scalability when printing therapeutic scaffolds, or building scaffolds with appropriate adhesion tissues. In situ bioprinters and printers cannot handle all of the problems listed above. Automated bioprinters are easy to operate and can create complex structures of varying thicknesses with minimal human intervention. The frequency at which the electric motor turns on and off can alter the extrusion rate.

Photo‐cross‐linkable hydrogels, such as GelMA for VML faults, may be printed in situ using the portable printer. By printing the GelMA scaffold directly on the skin, extra procedures and difficulties associated with hydrogel‐based scaffold implantation can be avoided. Hand‐held bioprinters, according to the scientists, may be used to alter muscle cells without affecting their capacity to survive and multiply. According to their findings, in situ constructed multinucleated myotubes can grow successfully.^80^

Similarly, Quint et al.^101^ worked on skeletal muscle regeneration and designed a bioink that uses controlled GelMA hydrogels and nano clay to perform controlled release of vascular endothelial growth factor (VEGF). Printing the ink at the mouse lesion site through a portable bioprinter showed that prepared ink improved muscle function and reduced fibrosis at the wound site.

#### 3.1.3 Cartilage regeneration

Cartilage is an aneural, avascular, and alymphatic tissue that its injuries' primary symptom could not be sensed^102^, ^103^ due to the fact that it has a sparse population of a chondrocyte, possessing the ability to regenerate post injury is prevented.^104^ On the other hand, cartilage injuries cause loss of function, significant pain and osteoarthritis (OA).

OA is the most prevalent long‐term degenerative cartilage disease, resulting in inflammation and cartilage breakdown. There is a lack of efficacy in current therapeutic therapies for cartilage abnormalities, such as autologous chondrocyte implantation and periosteal grafts. Other treatments include mosaicplasty, micro‐fracture, and mosaicplasty. For the time being, there are no therapeutic therapies that can replicate normal, mechanically and cellularly sound hyaline cartilage that is capable of withstanding everyday shear and compression.^79^ Attempts to use chondrocytes and MSCs in damaged regions of the body failed owing to a lack of nutrition and structural support needed.^105^

**FIGURE 10:**
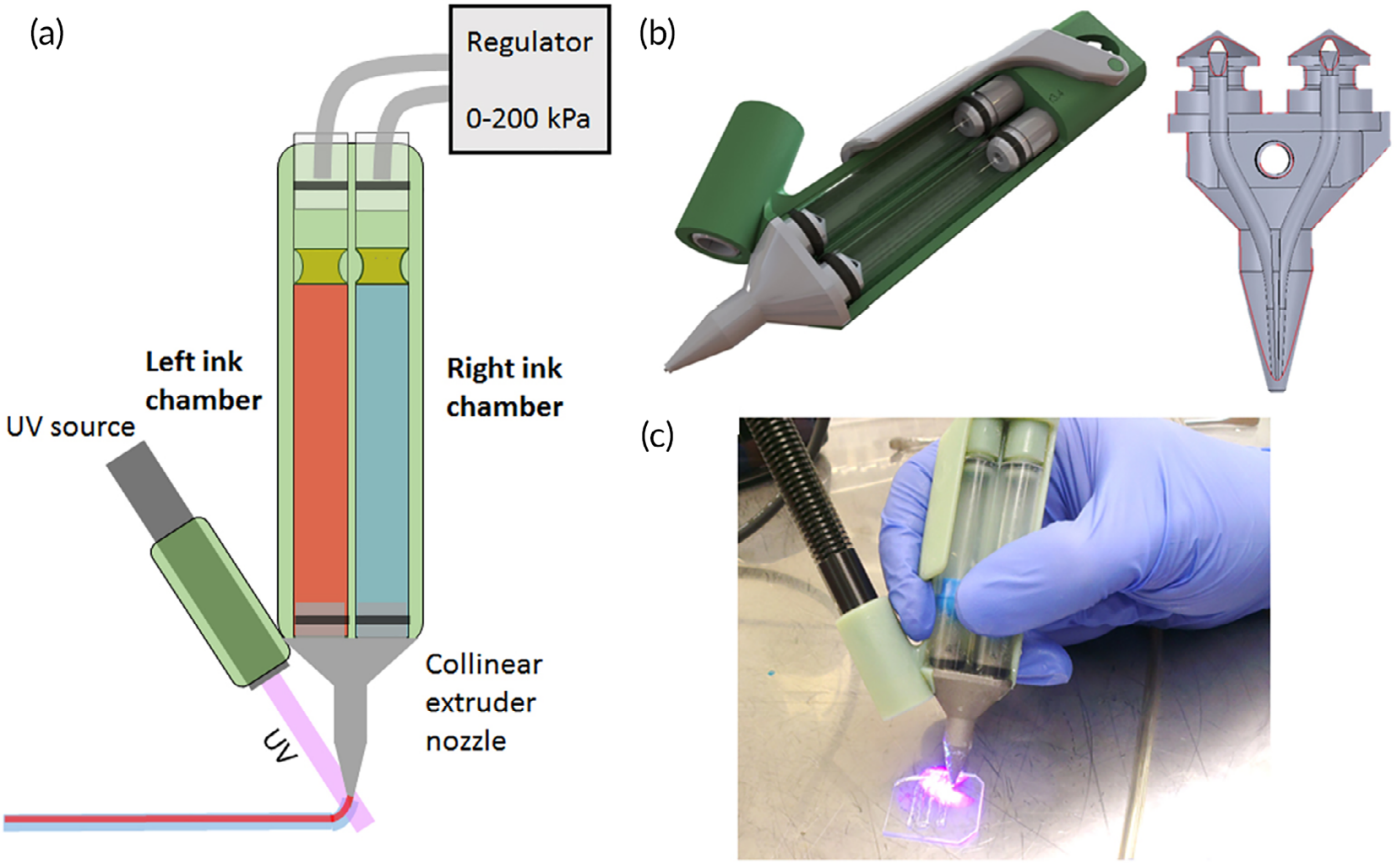
Biopen's design and application. (a) The portable Biopen's schematic. The pneumatic mechanism separately regulates extrusion through two ink chambers (left and right). Two foot pedals linked to pressure regulators control the extrusion process. A third foot pedal is used to activate the UV curing system. (b) On the left, a 3D CAD model of the Biopen's 3D‐printed components; on the right, a CAD model of the extrusion nozzle. (c) A picture of a Biopen that has been produced. *Source*: All pictures were reprinted with permission from reference 60

Recent advances in TE have enabled the treatment of cartilage injuries by implanting a biomaterial scaffold to fill in a gap and stimulate new tissue development. Another debridement step is needed to remove extra fibrosis tissue around the defect if using a TE method for fabricating the scaffold to fill the defect. As a result, the flaw and the constructed scaffold are out of alignment. Bioprinting may be able to repair osteochondral defects despite the disadvantages of implanting a manufactured scaffold. Connell et al.^60^ created an in vivo osteochondral repair device called a Biopen that is portable and uses pneumatic extrusion (Figure 10a). The nozzle's design allowed for collinear bioprinting of several bioinks (Figure 10b). The ability to regulate the printed structure geometrically and create compositional gradients is enabled by applying separate extrusion pressures to each chamber.

According to the authors, Biopen has several advantages over robotically manipulated clinical bioprinters, including the following: it enables surgical sculpting of the substitute tissue to achieve the desired structure, increased surgical dexterity enables deposition within crevices or under overhangs in tissue, it is easily transported into and out of the clinical field, it is easy to sterilize and maintain sterility, and it is less expensive than conventional methods. It may be used to encapsulate and transport human stem cells with a high rate of survival (>97%) following printing.^60^

Additionally, one of the main advantages of Biopen is the capability to 3D print numerous layers in real time utilizing a variety of biomaterials and cells to reconstitute diverse tissues by changing cartridges. Additionally, Biopen procedures were conducted without the doctors' or supporting company's particular training. Divide the ink cartridges, extrusion pistons, and extrusion nozzle of the Biopen that are directly in contact with cells for sterilization.^83^

An in vitro study investigated the high survivability of human adipose stem cells (hASCs) in a week after being printed along with a gelatin–methacrylamide/hyaluronic acid–methacrylate (GelMa + HAMa) hydrogel, through *Biopen* device.^60^ The authors prepared a mix of GelMA 10 wt%, gelatin 3 wt%, and HA 1 wt% hydrogel. They could minimize the cure time (1 s) by decreasing the extrusion speed of the bioink and increasing the exposure to material or light. The primary mission of this work was to design a bioprinter for printing hydrogels containing cells at the site of injury and simultaneous curing at the site (Figure 10c). The *Biopen* developed in this study directly deposits bone and cartilage scaffolds with or without the cells during the surgical process.^60^

**FIGURE 11:**
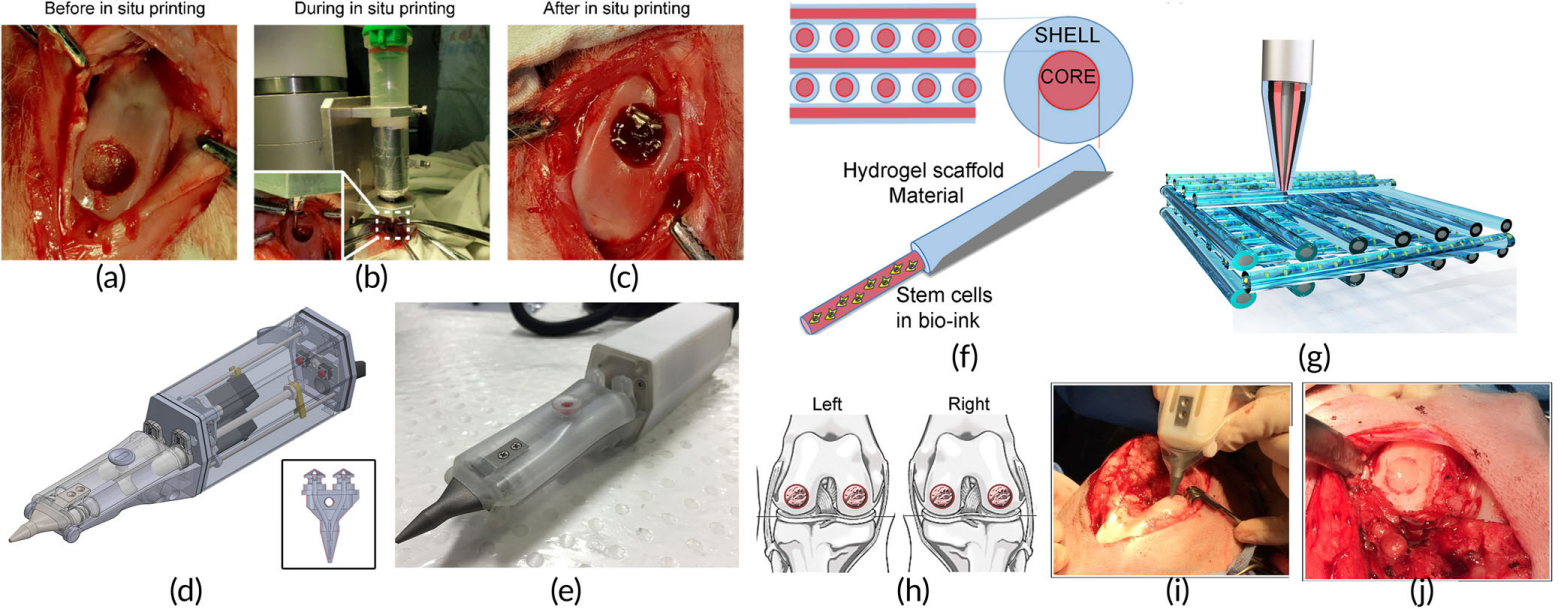
(a–c) In situ 3D bio‐printing applied to a rabbit's knee joint. *Source*: reprinted with permission from reference 59 . (d) The Biopen's design, which exhibits two distinct chambers controlled by a motor. The printing nozzle (insert) is linked to the two chambers, allowing for coaxial printing of the two distinct bioinks in a core/shell distribution. (e) A picture of the Biopen. (f) Core/shell distribution representation. The photo‐initiator VA086 for UV photocuring is included in the GelMa and hyaluronic acid methacrylate hydrogel (HAGelMa) in the shell, which provides mechanical support to the printed biomaterial while also protecting the inner core. Photo‐initiator is not present in the HAGelMa in the core, but it does contain mesenchymal stem cells. (g) A crisscross pattern is used to represent the multiple layer 3D‐printed block. (h) Both stifle joints have a full‐thickness chondral deficiency in the weight‐bearing region of the medial and lateral femoral condyles. (i) Photographs of the Biopen in use taken during surgery. (j) A bio‐scaffold is used to fill the defect, which is then covered with fibrin glue spray. The circular defect is evident, and it is macroscopically entirely filled while retaining the femoral condyle's apparent curvature. *Source*: (d–j) reprinted with permission from reference 52

Ma et al.^84^ developed a six‐degree‐of‐freedom robot‐based in situ 3D printing technology. They utilized a robotic bioprinter to repair grade IV cartilage lesions in their study, following the worldwide cartilage repair society's classification system (ICRS). Their bioink was composed of hyaluronic acid methacrylate (HAMa) and acrylate‐terminated four‐armed polyethylene glycol (4‐Armed PEG‐ACLT), which acted as a cross‐linker and was also capable of entirely covering the violation area and preventing cartilage degradation after 12 weeks (Figure 11a–c).

Bella et al.^52^ studied the full‐thickness cartilage defect model in sheep. They utilized HAMa‐GelMA hydrogel bioink infused with MSCs, which was covered with fibrin glue spray before being filled in. They engineered a hand‐held bioprinter (*Biopen*) to co‐axially print the two different bioink in a core/shell distribution (Figure 11d–g). They reported that the Biopen could be used in multiple applications, such as wound skin regeneration and pulp dental disease treatment.

Using the nozzle's unique design, the researchers were able to perform single‐session surgery on sheep's knee joints, depositing cells and the scaffold directly into the cartilage lesion. The results were promising. Two bioinks extruded from separate cartridges in a core–shell method during coaxial bioprinting, so the core bioink may contain stem cells and the shell bioink is cured by the photo‐initiator to process mechanical strength in the bioink. The outcomes investigated that the 3D‐printed scaffold had better characteristics than control groups and showed a novel formation of hyaline‐like cartilage via an in vivo study (Figure 11h–j).

**FIGURE 12:**
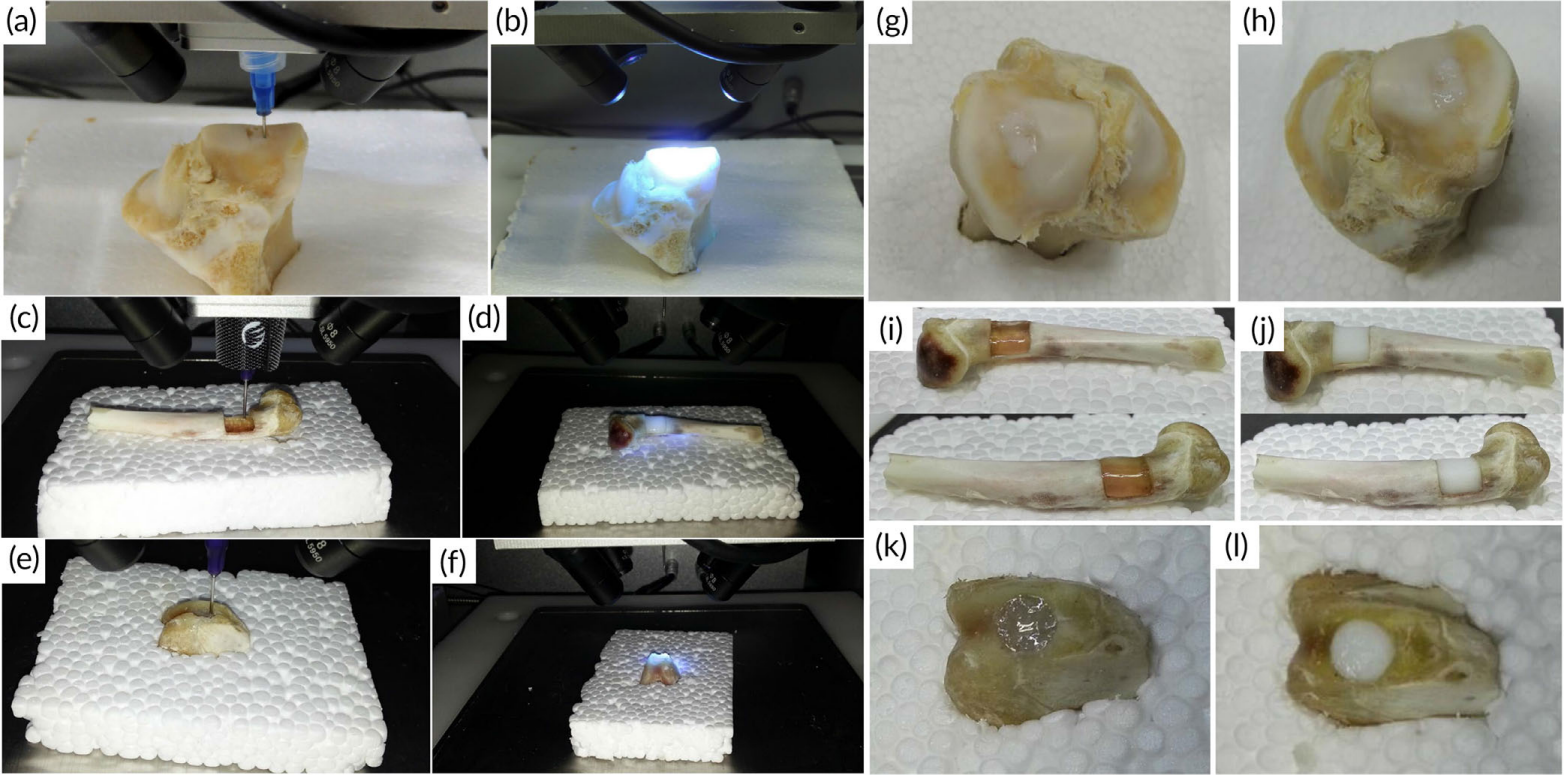
On chondral, bone, and osteochondral abnormalities, a 3D bioprinting and photopolymerization process was used. (a) In situ 3D bioprinting using m‐HA hydrogel to repair a chondral lesion. (b) UV light exposure of printed m‐HA hydrogel (c) in situ 3D bioprinting with alginate hydrogel to repair a bone defect. (d) UV light exposure of printed alginate hydrogel. (e) In situ 3D bioprinting with alginate hydrogel to repair an osteochondral lesion. (f) UV light exposure of printed alginate hydrogel. (g,h) Before and after photopolymerization, the color of the m‐HA hydrogel used to repair the chondral defect was milky white. (i) Before photopolymerization, the alginate hydrogel used to repair the bone defect was transparent. (j) After a few seconds of exposure to UV radiation, the hue of the alginate hydrogel became milky white. (k) Before photopolymerization, the alginate hydrogel used to repair the osteochondral lesion was transparent. (l) After a few seconds of exposure to UV light, the hue of the alginate hydrogel became milky white. The chondral, bone, and osteochondral deficiencies were all completely repaired. *Source*: All figures were reprinted with permission from reference 85

Li et al.^85^ combined 3D scanning and in situ bioprinting technologies to regenerate cartilage and bone defects. In their research, they modeled three flaws, such as substantial long‐bone segmental defects, free‐form femoral condyle fracture, and ICRS‐grade IV chondral lesion (Figure 12). It was discovered that using bioink for the treatment of cartilage disease, a tiny molecular molecule was effective. Since HA has superior features of biocompatibility, biodegradability, viscoelasticity, and nonimonogenesity compared to other potential cartilage printing bioinks, it was chosen suitably.

They utilized UV‐polymerized sodium alginate and polyethylene (glycol) diacrylate (PEGDA) to mend the bone deficiency. Hydrogels such as alginate are frequently utilized in 3D bioprinting as bioink because of its exceptional performance, such as appropriate cell attachment, printability, biocompatibility, biodegradability, low toxicity, and moderate gelation. PEGDA is also utilized in TE and targeted drug release as a drug carrier and small molecule due to its low degradation rate, noncellular adhesion characteristics and tunable mechanical properties.^84^ To make bone and cartilage scaffolds stronger, PEGDA has been utilized to carry tiny molecules including bone‐morphogenetic protein 2, collagen, and oligosaccharides. This increases the scaffold's flexibility as well.^106^

**FIGURE 13:**
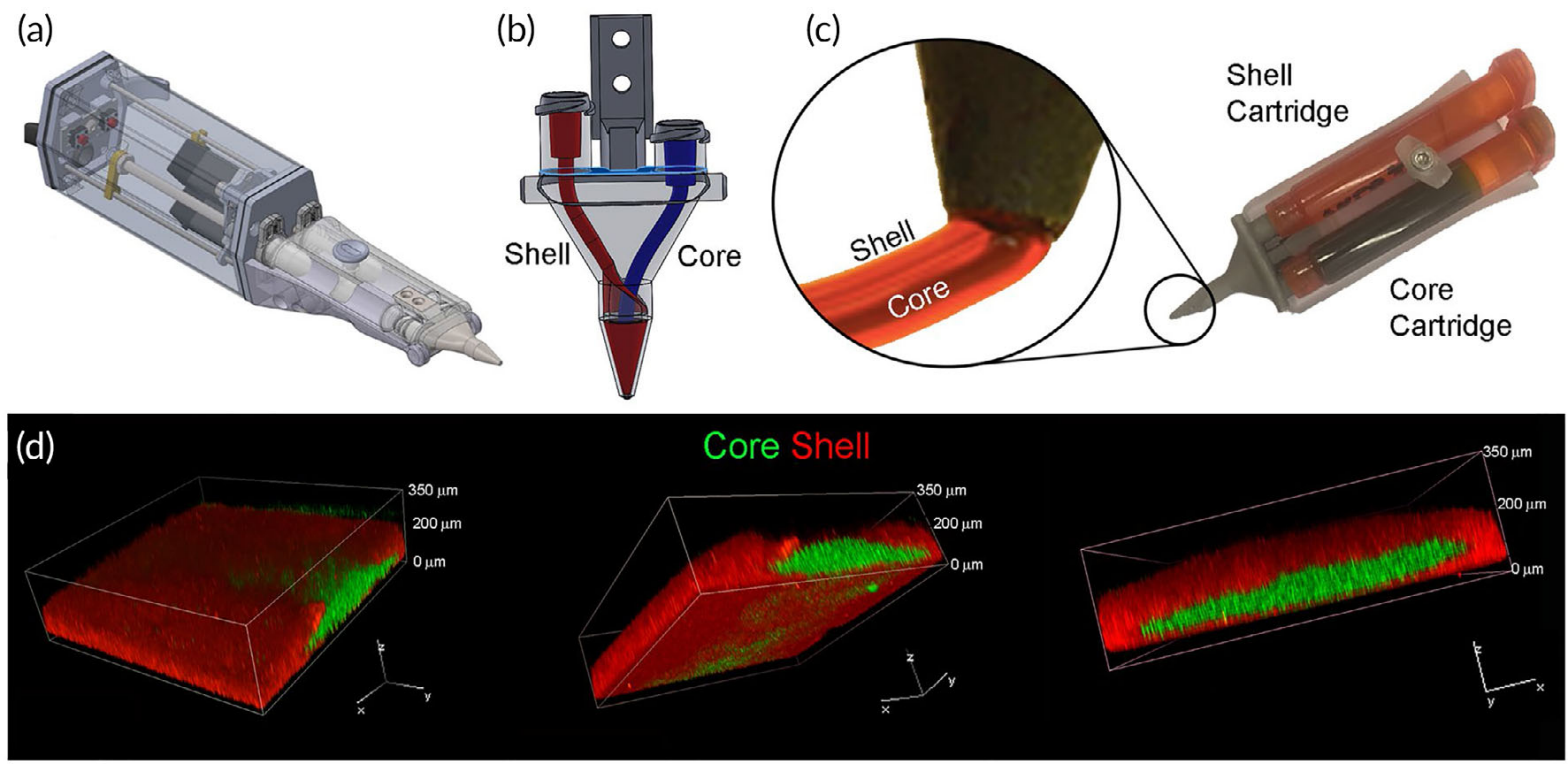
3D printing of the core and shell using co‐axial extrusion. (a) The 3D co‐axial hand‐held printer is depicted schematically. (b) The co‐axial nozzle is depicted schematically. (c) Illustration of the core and shell loading cartridges in the printer, showing relative magnification of the nozzle during co‐axial deposition. (d) Confocal pictures of a core/shell printed sample tagged with fluorescent beads, rendered in 3D. The Shell (GelMa/HAMa + 0.1% lithium‐acylphosphinate) is represented in red, while the Core (GelMa/HAMa) is displayed in green. The screen displays the same image in three distinct orientations, which represents a 3D rendering of overlaid green and red channels. *Source*: All pictures were reprinted with permission from reference 82

Duchi et al.^82^ prepared a co‐axial bioprinter and used it to the healing of cartilage defects by evenly depositing GelMA and HAMa hydrogel and encapsulating ADSCs‐derived chondrogenic cells from adipose‐derived mesenchymal stem cells. More than 90% of the stem cells in the Biopen (Figure 13) were able to survive and sustain their multiplication capacity, which was verified by the researchers. On the other hand, since cell viability is critical for this treatment, they used the core/shell process that increased cell viability and accelerated repair of osteochondral lesions.

Generally, the core/shell portable 3D bioprinting strategy could rapidly produce high modulus scaffolds with high cell survivability and have a potential for in situ surgical cartilage engineering use. In addition, the authors pointed out that the fabricated portable 3D printer might be used to implant co‐axial cell‐containing scaffolds in surgery, particularly for the restoration of musculoskeletal tissue.^82^

Xu et al.^81^ prepared a unique injectable stem cell‐laden gelatin supramolecular hydrogel with a new approach, host–guest macromer (HGM) osteochondral regeneration. They demonstrated that in situ injection of the HGM hydrogel improves chondrogenesis in rat knee cartilage defect. They also showed that this hydrogel could be used as a drug and encapsulated cell carrier to enhance the treatment time and quality.^81^ Furthermore, Onofrillo et al.^83^ used the Biopen for in situ bioprinting of GelMA and HAMa and human‐derived mesenchymal stem cells (hADSCs) into chondrogenic media for 8 weeks, then analyzed this scaffold for treatment of cartilage patients. In this method, by designing Biopen as nuclear/shell instrument, they prevented the transfer of extrusion pressure to the cells. Also, they increased the cell viability by preventing direct contact of photo‐initiator and toxic chemical by‐products with the cells. Thus, they successfully demonstrated that this co‐axial extrusion Biopen was a suitable device for surgical treatment since it has increased cell viability during bioprinting and produced hyaline‐like cartilage.^83^

The in situ bioprinting technology introduced a novel strategy for printing the biologically active construct in a patient‐specific manner directly at the injury site in a considerably shorter time. For instance, in situ printing of a clinically conformant liver defect is an accessible target in soft tissue regeneration. The MRI obtained from patients is used to create a CAD image of the injured area that requires reconstruction. Using that image as a guide, a multihead bioprinter fabricated a 3D construct via different bioinks, possessing multiple cell types and growth factors for exactly recapitulating the structural and biological (cell‐synthesized ECM) component of the lost tissue, which was directly printed onto the damaged liver of the patient.^107^

### 3.2 Hard tissue regeneration

#### 3.2.1 Bone regeneration

Although surgical procedures have improved significantly in recent years, bone tissue regeneration after injury is still difficult to achieve structurally and functionally.^108^ The conventional treatments of critical‐sized bone defects included culturing bone tissues from autografts or allografts and implanting the cultured bone tissue into the defected area are going on.^109^ Autograft approaches are considered the gold standard in bone fracture treatment. In spite of the fact that autografts have been around for a long time, they have a number of disadvantages, including donor site morbidity, sensitivity, deformity, muscle weakening, infection, and persistent discomfort.^86^ Foreign‐body implants, which fill in the bone deficiency and offer structural support and mechanical strength, are an additional treatment option for problems.^110^ However, foreign‐body implants do not offer permanent treatment for children and middle‐aged individuals since they are associated with poor functional and esthetic consequences.^111^ Bone tissue engineering (BTE) provides an alternative approach for generating new bone tissues and treating bone diseases.^112^ The major difficulty of BTE is to produce natural bone tissue‐like mechanical strength and sufficient porosity for vascularization, which is categorized as hard tissue.

Bioprinting research has looked at bone tissue.^90^ Open fractures in the skeletal system can be treated using 3D scanning and 3D bioprinting.^89^ Because of the high 3D scan resolution (up to 5 m), the skeletal system may be successfully rebuilt using this technology. The portability of portable printers makes it possible for surgeons to obtain very precise 3D digital models in the operating theater in a matter of minutes.^12^ Laser‐assisted bioprinting (LAB) has been shown to be an appealing method for bone tissue bioprinting in situ. According to the results of their research, the LAB method may be utilized to print MSCs within collagen and nano‐hydroxyapatite in a mouse Calvaria defect model for bone tissue regeneration.

Their study showed the first usage of the in situ LAB approach in a critical‐sized bone regeneration via a well‐defined pattern along with proving the safety of the LAB approach bioprinting in vivo. They also showed that different cell arrangements at the site have a significant effect on bone tissue regeneration. This effect showed the progress of the in situ bioprinting method for building tissue. Furthermore, they demonstrated that LAB with in situ bioprinting technology is suitable for bone repair and random‐shaped cartilage defects.^12^

An in situ extrusion bioprinting for cartilage and bone deformities from the Cohen et al.^113^ work was successfully reported. These matrices were printed on an osteochondral defect in order to assess the potential for bone and cartilage tissue regeneration in their study. Their study demonstrated the feasibility of the in situ reconstruction system with great precision due to the geometric feedback and also progressive path planning techniques, while biomaterial selection for scaffolding and its applicability in the human body are significant challenges yet.

Furthermore, as reported previously, Li et al.^85^ showed 3D scanning and bioprinting to have the ability to fix skeletal and cartilaginous problems. Using in situ printing to quickly and accurately obtain a 3D digital model, a new treatment method for osteochondral injuries has been developed. This technology might be more effective and useful in treating difficult, unique abnormalities. A random‐shaped chondral defect, a cuboid‐shaped osteochondral defect, and a cylindrical‐shaped bone defect were all examined to see whether they could be repaired in situ. An alginate‐based hydrogel was employed as a bio‐ink in the aforementioned situations to help regenerate osteochondral lesions. In addition, the chondral defect was repaired using a hydrogel based on modified sodium hyaluronic acid (HA). For the first time, chondral and osteochondral functional restoration has been attempted using computer‐controlled layer‐by‐layer synthesis of different hydrogels based on extrusion‐based 3D in situ bioprinting and photopolymerization.

**FIGURE 14:**
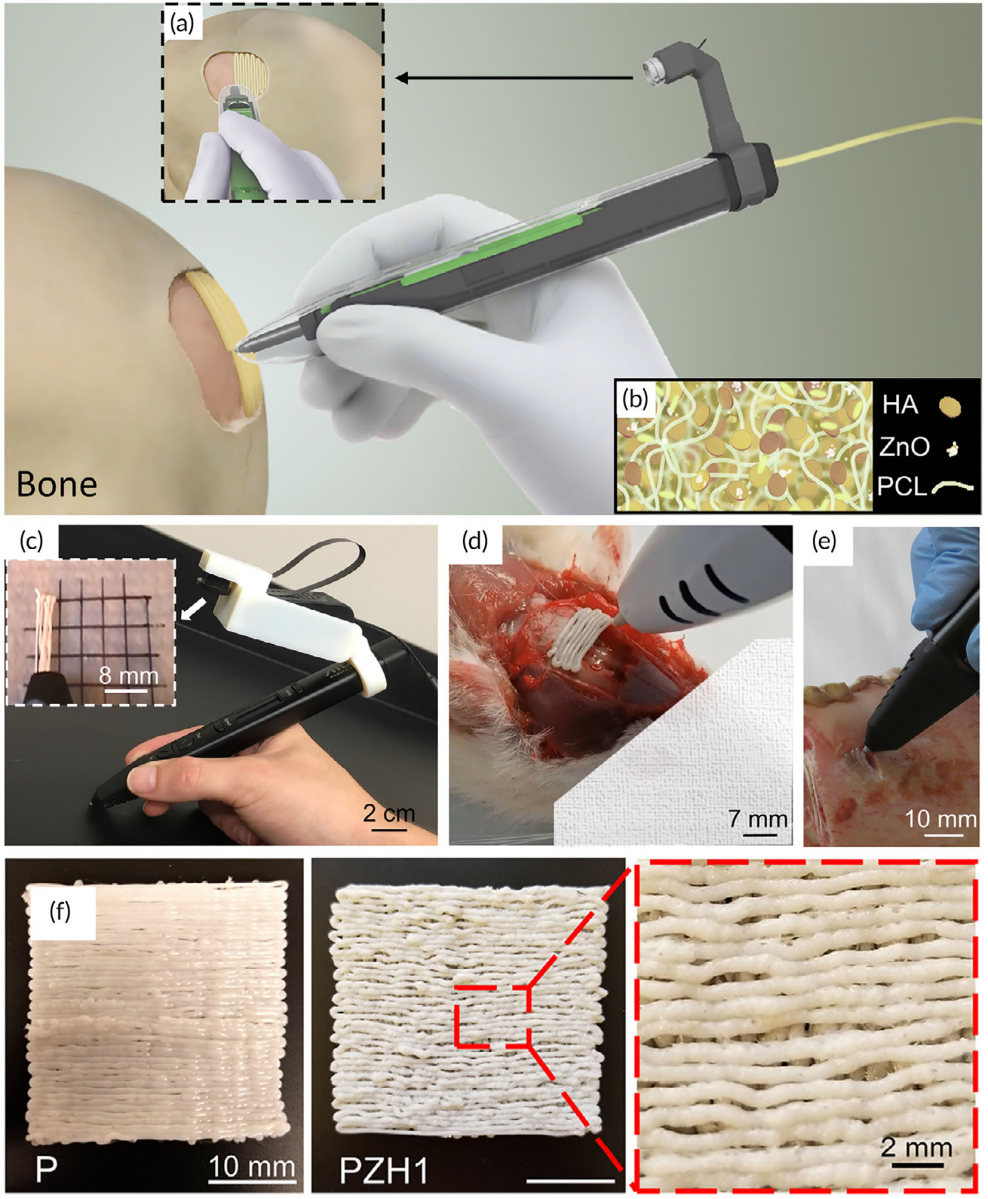
Using a hand‐held instrument, in situ printing of manufactured filaments. (a) A hand‐held printer with an integrated camera prints a composite polymeric system comprising PCL, HAp microparticles, and ZnO nanoparticles (b) a schematic of the material composition (c) A representative image of the hand‐held melt‐spun 3D printer that was used to print the filaments, as well as the tiny integrated camera that was utilized to monitor printing quality on the go. Ex vivo printing of scaffolding materials into a flaw created within a fresh pig jawbone. (d) ex vivo free‐standing printing structure on the calvarial bone defect in a euthanized rat. (e) Ex vivo printing of scaffolding materials into a defect formed inside a fresh porcine jawbone. The scaffolds remained attached to the bone and did not come loose. (f) Representative pictures demonstrating the printing quality utilizing various formulations. Pores are created as a result of the formed defects, which are believed to facilitate cellular infiltration. *Source*: All pictures were republished with permission from reference 86

Mostafavi et al.^86^ were able to design fused portable printers called *Pen Bone* and insert the material ink directly into the cavity (Figure 14). In their work, scaffolds were prepared from a polycaprolactone (PCL)‐based ink compound with zinc oxide nanoparticles and hydroxyapatite (HAp) microparticles. HAp, which has a significant contribution to hydrophobicity, has been used to improve protein absorption. Also, zinc oxide nanoparticles were used to inhibit bacterial growth on the scaffold surface. Printed scaffolds showed that they have an appealing support structure for MSCs.

#### 3.2.2 Dental regeneration

**FIGURE 15:**
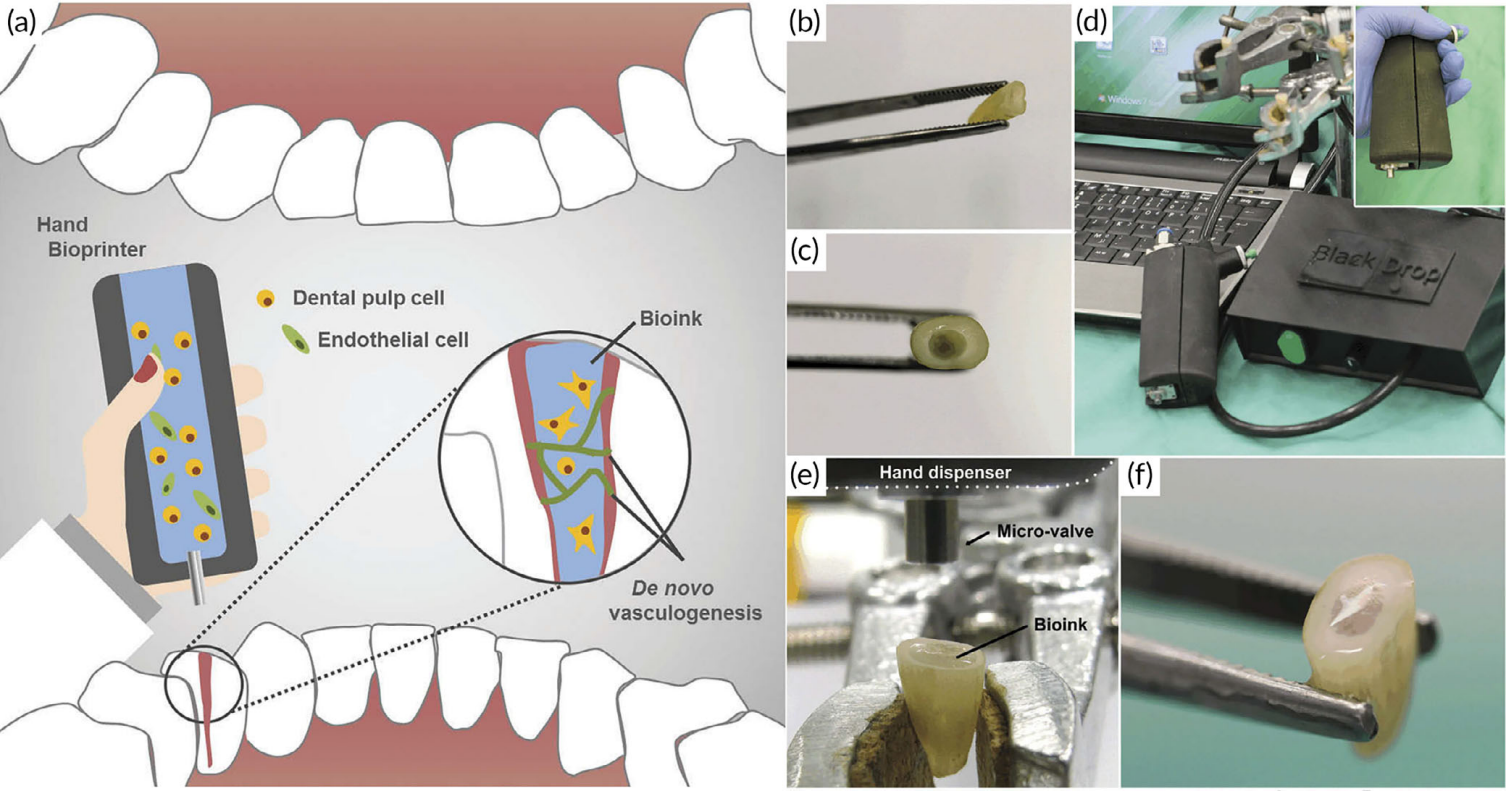
Using a hand‐held bioprinter, in situ bioprinting of human teeth dental pulp tissue. (a) Design for hand‐held in situ bioprinting of cell‐loaded bioinks for de novo dental pulp tissue vasculogenesis. (b, c) Macrographs of cleaned root canals in prepared human teeth. (d) Setup for hand‐held bioprinting, which includes a hand‐held device with cartridge and microvalve, as well as a control unit. (e, f) Macrographs of human teeth following in situ hand‐held bioprinting with 0.2% COL–0.5% AG bioink and showing no indications of hydrogel contraction. *Source*: All pictures were reprinted with permission from reference 87

The advent of novel portable bioprinting technology has attracted many scientists' attention in various scientific fields, including dentistry.^114^ Campos et al.^87^ prepared a printable agarose (AG), collagen type I (COL1), and fibrin hydrogels in 0.5 wt% FIB, 0.3 wt% COL1, 0.2 wt% COL1, and 0.5 wt% AG amounts. They stimulated root canal vascularization using human primary dental pulp cells and endothelial cells (Figure 15). Their research looked at the performance of vascular tube creation using two different types of bioinks, including printable and nonprintable (containing 5% FIB and 3% COL) (0.2% COL and 0.5% AG). To help with tissue structure, they worked to create vascular tubes out of printable bio‐inks. In comparison to casting or pipetting methods, they discovered that in situ bioprinting of injectable inks had numerous benefits. Pipette casting does not fill in some cases or overfill inside the canal due to the limited volume inside the root canal. Also, in pipette casting, viscous liquids enter the tooth cavity, which prevents the complete penetration of the root canal due to the presence of air bubbles in its structure. However, in the injectable bioprinter method, air bubbles are prevented from forming by increasing the material transfer speed.^87^

## 4 CHALLENGES AND PERSPECTIVES

### 4.1 Challenges

As mentioned previously, traditionally, bioprinting procedure consists of three separate and dependent steps. The first step is acquiring medical image through CT or MRI software, afterwards, calculation of size and shape of printed constructs, based on the resulted images. Fabricating 3D construct from prepared model requires proper bioprinting approach, suitable bioinks and biomaterials with printable rheology and mechanical performance. The final step is maturation of printed tissues in various strategies, including maturation in bioreactor in vitro, integration with healthy tissues in vivo for accurate maturation, and utilized for in vitro applications.^15^ However, all the steps are critical in the procedure, the printing step is the most complicated as it is vital to fabricate biological constructs with suitable characteristics.

Although, the implantation of fabricated structures has faced to several issues associated to printed tissues integrations with environmental tissues, namely, production of fitted construct for injured area, surgical removal of dead tissues from defect location and risk of contamination. To tackle these limitations, in situ bioprinting technology emerged to directly print and deposit constructs at the patient's defect site.^115^, ^116^

Campbell et al.^117^ presented this novel concept of in situ bioprinting technique for the first time, when they investigated inject strategy for bone regeneration in the rat Calvaria defect model. Since the first report of in situ bioprinting in 2007, several investigators have studied two different approaches: the first approach depend on fundamental research to develop in situ bioprinting technology (such as discovering the desired bioinks and developing bioprinting procedure) and another one involves this technology application in various animal disorder models including cartilage, bone, cartilage, skin, dental, and skeletal muscle in vivo defect models, as reported previously. An early report described the combination of geometric feedback‐based strategy with proper biomaterials for appropriate in situ chondral and osteochondral injuries repair.^118^, ^119^ The ideal in situ bioprinter should scan the exact area of defect to detect the injured site and produce matched printed tissues.

Although, the maturation of printed tissues utilizing bioreactors in vitro, modifications in size and form and evaluation of cultured tissues safety as preimplantation operations should be done before their in vivo application administration.^120^ In situ 3D bioprinting strategy has been established to overcome these limitations by directly printing on damaged locations or defects and using cellular microenvironment potential to remain postimplantation survival and functionality of printed tissues.^121^

However, several challenges remain unsolved and have to be tackled. Some tissues in situ bio‐production needs a high amount of living cells; thus, a cell culture method will predominantly produce sufficient cells before surgery. However, developing an effective in vitro cell culture expansion method (to produce a high number of viable stem cells with stemness and proliferative properties) and acquiring the ethical approval for embryonic stem cells (ESCs) restrict their medical applications. On the other hand, the post‐printing maturation of grafts requires reliance on structural stability, vascularization, immune system reactions, stem cell proliferation and differentiation and biodegradation kinetics of the bio‐printed construct.^107^, ^122^

Current investigation has reported various external cues for gelation of bioinks that are not biocompatible, as these studied cues cannot be applied within the patient body. Another limitation in the maturation of printed tissues is an insufficient vascular network to provide nutrient molecules and oxygen to deep tissues.^123^ However, it is proposed to solve vascularization limits before the clinical development of in situ bioprinting fabrication.

Additionally, biocompatibility between expanded stem cells and biomaterials, adequate cell proliferation, and correct differentiation, which supplies the capacity to stem cells, restore the natural ECM. The biodegradation rate of printed biomaterials should match with the ECM production rate by seeded stem cells. The biodegradation rate control in vivo has multiple problems. Other post‐printing challenges, including rapid solidification, combining environmental healthy tissues, and make sterile conditions are vital considerations in tissue biofabrication and maturation processes.^48^, ^124^ The improvement and successful therapeutic interventions are expected to provide more cost‐effective and user‐friendly treatment. Therefore, in situ bioprinting integration with robotic system technology prepares a more accessible technique for surgical operations. Besides, various advanced imaging instruments would be combined with robotic systems to evaluate the damaged area to fabricate printed tissues in vivo. All these progress will accelerate in situ bioprinting technology translation from the bench to the bedside.^9^, ^125^

### 4.2 Perspectives

As mentioned previously, multiple 3D bioprinting technologies have been appeared and applied to diverse medical applications, ranging from evaluating of the cellular process to regenerating tissues, organoids, and organs for in vivo implantation.^126^ The remarkable ability of 3D bioprinter technologies makes a promising strategy for tissue damages, even in situ and has been displayed to transfer cells, biomaterial, and biomolecules to target damaged locations in a site‐specific manner.^127^ 3D bioprinting technology can be integrated with other advanced approaches such as cell therapy, and microfluidics to fabricate automatic manageable printed devices to reconstruct the diverse biological structures and essential composition of targeted tissues as well as increasing drug discovery procedures and developing an effective treatment for genetic disorders.^128^, ^129^

3D printing strategies, which are commonly dependent on the printed biodegradable cell‐free constructs, are eventually applied for implantation in the TE field. In this top‐down method, the cells as key factor of tissue regeneration is only seeded at the end of 3D printing procedure, some hurdles, such as inaccurate cell distribution over the printed grafts occurred, which led to failing appropriately regeneration due to the incorrect ECM ultrastructure with loss sufficient cell‐ECM interactions. Therefore, 3D bioprinting appeared as bottom‐up approach to create artificial printed tissues or organs in a layer‐by‐layer manner through critical tools, including proper biomaterials, various cell types, and growth factor molecules.^2^, ^130^

Recent studies show the great potential of in situ 3D bioprinting technology as a feasible regenerative method in TE.^131^ It has been suggested that the natural cellular microenvironment in the patient body can promote maturation and merging of printed grafts to injured tissues or cells due to different and huge chemical and physical signals.^121^ Moreover, in situ printing procedures with less invasive operating procedures can be introduced as a user‐friendly technology for surgeons that increase outcomes therapeutically.^132^

Also, some successful efforts reveal the future perspectives of the portable hand‐held bioprinters' applications. Researchers created an easy‐to‐use syringe for the hydrogel that could chill the gel quickly before application on the front lines.^133^ An ice pack‐like chamber in the syringe that contains calcium ammonium nitrate crystals was used. Within 30 s of introducing water to the chamber, the crystals begin to activate and chill the hydrogel to its operational temperatures. As a result, they could modify the hydrogel delivery device so that it not only swiftly cools the hydrogel but also retains it at that temperature, providing a 10‐min window to fill penetrations in the eye. This is like caulking a bathroom seal in terms of simplicity. Hence, injuries to the eyes caused by piercing objects will heal faster with the new seal and delivery system.^133^ The portable hand‐held bioprinters could promote this healing process with the homogenous extrusion of the material from the nozzle.

Moreover, surgical sealants have been used for sealing or reconnecting ruptured tissues but often have low adhesion, inappropriate mechanical strength, cytotoxicity concerns, and poor performance in biological environments.^133^ To address these challenges, researchers engineered a biocompatible and highly elastic hydrogel sealant with tunable adhesion properties by photo cross‐linking the recombinant human protein tropoelastin.^134^

In rodents, the subcutaneous implantation of the methacryloyl‐substituted tropoelastin (MeTro) sealant demonstrated low toxicity and controlled degradation.^134^ All animals survived surgical procedures with adequate blood circulation by using MeTro in an incisional model of artery sealing in rats, and animals showed normal breathing and lung function in a model of surgically induced rat lung leakage. In vivo experiments in a porcine model demonstrated complete sealing of severely leaking lung tissue in the absence of sutures or staples, with no clinical or sonographic signs of pneumothorax during 14 days of follow‐up. The engineered MeTro sealant has a high potential for clinical applications because of superior adhesion and mechanical properties compared to commercially available sealants, as well as the opportunity for further optimization of the degradation rate to fit desired surgical applications on different tissues.^134^

The practical idea of sealing or reconnecting ruptured tissues through employing the portable hand‐held bioprinters is another perspective that could promote surgical and suturing operations. High‐pressure bleeding in pigs' hearts can be stopped with a novel bio‐glue, an experimental adhesive gel activated by a flash of light. According to the World Health Organization (WHO), more than 234 million procedures are performed each year around the world.^135^ According to a previous study, surgical suturing is particularly challenging when dealing with sick, damaged, or tiny blood arteries.^136^ Fibrin Glue and Surgiflo, two commonly used surgical materials that effectively stop bleeding during surgery, take minutes to solidify and may necessitate additional stitching in some circumstances.^137^ Wounds and punctures of hearts, among the most difficult of surgical challenges, could also be sealed using only the bio‐glue, with no stitches.

Meanwhile, combining the portable bioprinters with other instruments could be applicable. Zhao et al.^138^ designed and fabricated an in situ bioprinting device that can print inside the human body utilizing a micro bioprinting platform attached to an endoscope. The platform was designed and built using printed circuit micro‐electro‐mechanical‐system techniques. The platform's viability was tested via a high‐precision control system and a suite of performance tests. Applying a stomach model, two‐layer tissue scaffolds were created, and human gastric epithelial cells along with human gastric smooth muscle cells were employed as bioinks in gelatin–alginate hydrogels to imitate the stomach's architectural shape. Cell viability and proliferation on printed tissue scaffolds were shown to be high after 10 days of cell culture, indicating that the cells were performing well biologically. The authors used an endoscope to insert the device into a transparent stomach model for the experiment.^138^ The prototype was ready to begin bioprinting on the stomach wall with epithelial and muscle cell‐laden gels, on arrival. It is possible that these 3D‐printed tissues could someday repair genuine ulcers in the digestive system because they retained their vitality and proliferated for 10 days. A significant step forward in treating stomach wounds could be made with this in situ bioprinting technology, as conventional therapies are typically slow and not consistently effective. The authors prospected that micro‐robots with cameras and other sensors could be developed in the future, allowing them to carry out more sophisticated tasks. The bioink kind of portable bioprinter is one of the most complex issues. Choosing a bioinject that can gel at 37°C is critical since multiple biomaterials liquefy at body temperature.

### 4.3 Commercialized hand‐held bioprinters

The promising results of the bioprinters and the countless advantages compared to conventional methods of fabricating TE scaffolds have attracted considerable attention.^139^ Of the foremost obstacles and challenges of bioprinters' commercialization is their high manufacturing cost. Over time, with innovative designs and bioprinters classification, some difficulties have been solved depending on their application.^140^ A $10.8 billion market is predicted for bioprinting, but it still faces significant commercial challenges due to the bioprinting process' optimization, which is currently not automated and requires manual operations in various steps, resulting in a slow processing speed and increasing the risk of error.^19^ Regulatory approval, insurance, hospital and medical regulations, and logistics are just a few of the major hurdles that must be overcome in addition to manufacturing that may be scaled up.^19^

Today, portable bioprinters have mostly removed this industry's previous limitations.^48^ As recent surgeries are being robotically and intelligently established, portable bioprinters can be a trustworthy option for minimally invasive and cost‐effective surgeries.^48^ Pereira et al.^141^ designed the first commercial bio‐printer in Germany and later commercialized it successfully. Commercially available bioprinting in situ printers have yet to be developed. Even so, a few commercially available robotic surgical systems, such as the MAKOplasty® knee replacement system and the da Vinci® surgical system, are now in use in the field of orthopedics (such as cardiac, thoracic, colorectal, and many others).^142^ They have demonstrated its efficacy in conducting the procedure with reproducibility and accuracy. In its infancy, in situ bioprinting may not completely replace traditional TE methods, but it will certainly enhance them.

**FIGURE 16:**
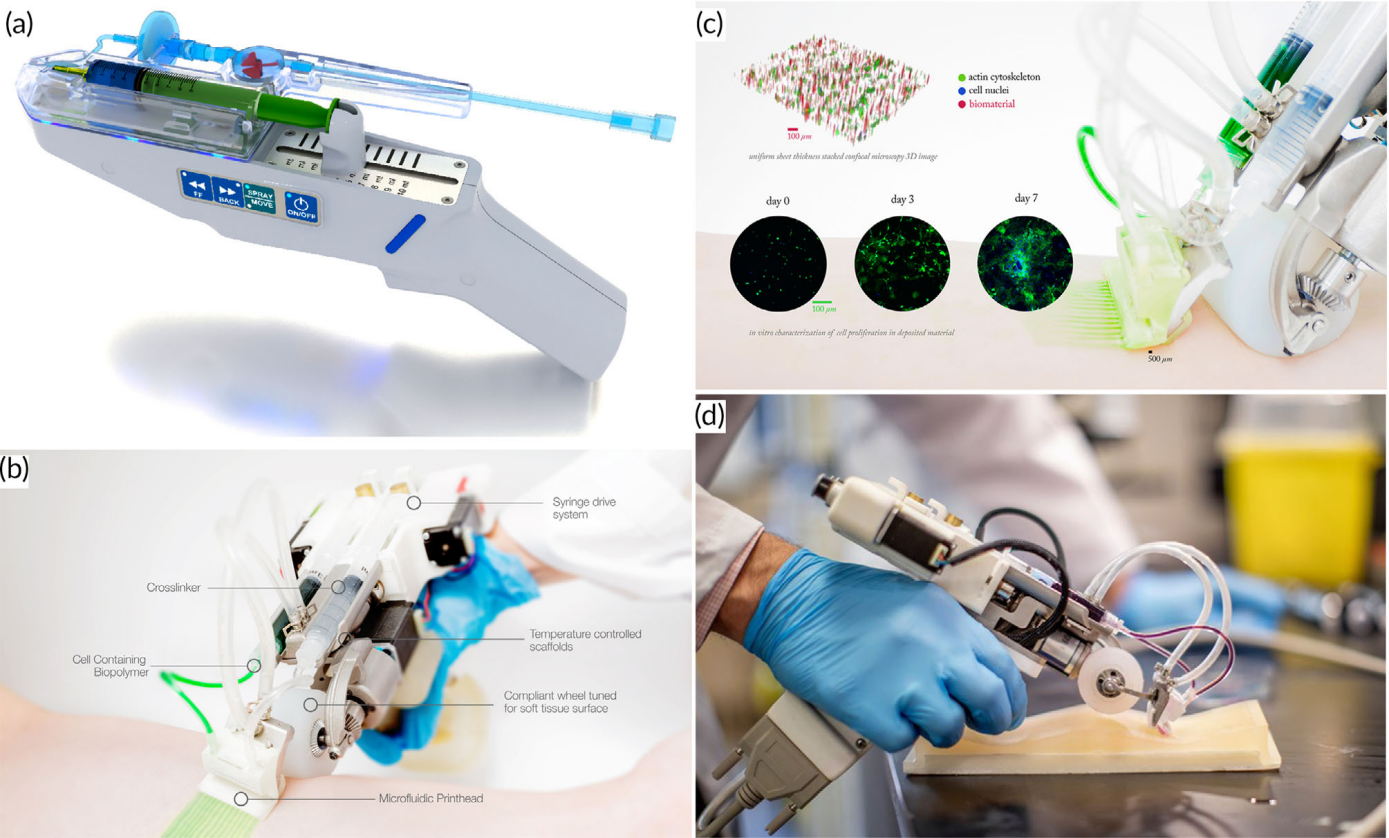
(a) RenovaCare's CellMist™ System and SkinGun™ spray device. *Source*: reprinted with permission from reference 145 (b) Portable bioprinter for skin TE. *Source*: reprinted with permission from reference 146 .(c) Portable bioprinter for wound healing, and (d) the portable bioprinter has undergone numerous iterations and is fitted with a roller. *Source*: reprinted with permission from reference 53

Nowadays, portable bioprinters are available worldwide, manufactured by several companies, such as Duplo‐jet (Baxter, Austria),^143^ Vivostat (Vivostat, Denmark),^144^ and Skingun (Renovacare, USA) **(**Figure 16 **).** ^145^ A “Biopen” portable bio‐printer was developed at several company, which can print biomaterials with cells at the injury site. Commercially available portable bioprinters are manually controlled and do not have a computerized system, whereas, they are cost‐effective and portable.^11^

ReverTome^146^ is a new device that provides sterile conditions for treating extensive burns with uniformly deposit cell and biomaterial precursor at the wound site and accelerate wound healing. Direct cell delivery removes the restrictions on the use of autologous skin removed from the patient. It has assisted physicians significantly in treating serious burns, reducing pain, healing time, infection risk, and length of stay in the hospital. According to the explanations provided about portable bioprinters, kindly noted that the advantages of portable bioprinters, include low cost in manufacturing the device, as well as easy use at the site of the disease, simultaneous bioprinting of several bioprinters, easy movement of the device and no need for complex computer systems.

It is possible to design a method for putting cell‐friendly, but mechanically weak biomaterials directly onto the wound of a patient. This printer (ReverTome) was designed to print on huge, flat, well‐defined regions, but massive animal experiments revealed that it could only reliably print on tiny, flat, well‐defined areas in its first iteration.^146^ A flexible print‐head with two degrees of freedom was included to provide uniform deposition on the heterogeneous surfaces of the human body in the second and third‐generation designs, which positioned the wheel behind the microfluidic cartridge to promote large‐area bioprinting. The fourth‐generation utilized a compliant wheel to reduce stress on the wound bed during the bioprinting process. The print‐head components were redesigned to facilitate sterilizing between surgeries by making it easier to disassemble and reassemble the print‐head. The current design includes temperature control to accommodate a broader range of biomaterials, such as thermally cross‐linked proteins. The manufacturer incorporated ergonomically‐designed control mechanisms into the handheld printer's sleeker new form factor as an added bonus.

The portability of the bioprinter, the ease with which sterile microfluidic cartridges may be replaced, and the ability to pattern soft materials with high fidelity on physiologically relevant surfaces in a clinical setting are the competitive advantages of the novel design. It is impossible to manage the composition of biomaterials across a complicated human topography using other cell delivery methods, such as direct spraying or implantable scaffolds. Commercial bioprinters like RegenHu and EnvisionTEC are potential rivals.^147^ Even though these machines can print high‐resolution biomaterials, they are cumbersome, expensive, need a high level of technical expertise, and are primarily used for research.

## 5 CONCLUSIONS

The remarkable ability of 3D bioprinter technologies makes a promising strategy for tissue damages, even in situ, and has been displayed to transfer cells, biomaterial, and biomolecules to target damaged locations in a site‐specific manner. Bioprinters, and especially the last generation of them, portable hand‐held bioprinters, as a novel facility of fabricating engineered tissues, positively correlate with the ultimate goal of regenerative medicine, which is the restoration, reconstruction, and repair of lost and/or damaged tissue function. With the advent of portable hand‐held bioprinters, the obstacles and challenges of utilizing statistical bioprinters could be resolved. Their application in in situ printing promotes tissue regeneration and could widen the application of AM in regenerative medicine. Meanwhile, the challenges and perspectives of the portable hand‐held bioprinters demonstrate the bright future for researchers. As soon as the commercialization process of portable hand‐held bioprinters becomes widespread and available in hospitals, the patients would enjoy their merits in lowering the infections and the time to be hospitalized.

## CONFLICT OF INTERESTS

The authors declare that they have no conflict of interest.

## AUTHOR CONTRIBUTIONS

**Zahra pazhouhnia:** Conceptualization (equal); data curation (equal); investigation (equal); methodology (equal); resources (equal); visualization (equal); writing – original draft (equal). **Nima Beheshtizadeh:** Conceptualization (equal); investigation (equal); project administration (equal); supervision (equal); validation (equal); visualization (equal); writing – original draft (equal); writing – review and editing (equal). **Mojdeh Salehi Namini:** Investigation (equal); methodology (equal); resources (equal); writing – original draft (equal). **Nasrin Lotfibakhshaiesh Lotfibakhshaiesh:** Project administration (equal); supervision (equal); validation (equal).

### PEER REVIEW

The peer review history for this article is available at https://publons.com/publon/10.1002/btm2.10307.

## Data Availability

The data that support the findings of this study are available from the corresponding author upon reasonable request.
